# *Padi2/3* Deficiency Alters the Epigenomic Landscape and Causes Premature Differentiation of Mouse Trophoblast Stem Cells

**DOI:** 10.3390/cells11162466

**Published:** 2022-08-09

**Authors:** Noura N. Ballasy, Elizabeth A. Bering, Caroline Kokorudz, Bethany N. Radford, Xiang Zhao, Wendy Dean, Myriam Hemberger

**Affiliations:** 1 Department of Biochemistry and Molecular Biology, Cumming School of Medicine, 3330 Hospital Drive NW, University of Calgary, Calgary, AB T2N 4N1, Canada; 2 Department of Cell Biology and Anatomy, Cumming School of Medicine, 3330 Hospital Drive NW, University of Calgary, Calgary, AB T2N 4N1, Canada; 3 Alberta Children’s Hospital Research Institute, 3330 Hospital Drive NW, University of Calgary, Calgary, AB T2N 4N1, Canada

**Keywords:** PADI, histone citrullination, DNA methylation, trophoblast stem cells, trophoblast giant cells, epigenetic regulation

## Abstract

Histone citrullination is a relatively poorly studied epigenetic modification that involves the irreversible conversion of arginine residues into citrulline. It is conferred by a small family of enzymes known as protein arginine deiminases (PADIs). PADI function supports the pluripotent state of embryonic stem cells, but in other contexts, also promotes efficient cellular differentiation. In the current study, we sought to gain deeper insights into the possible roles of PADIs in mouse trophoblast stem cells (TSCs). We show that *Padi2* and *Padi3* are the most highly expressed PADI family members in TSCs and are rapidly down-regulated upon differentiation. *Padi2/3* double knockout (DKO) TSCs express lower levels of stem cell transcription factors CDX2 and SOX2 and are prone to differentiate into extremely large trophoblast giant cells, an effect that may be mediated by centrosome duplication defects. Interestingly, *Padi2/3* DKO TSCs display alterations to their epigenomic landscape, with fewer H3K9me3-marked chromocentric foci and globally reduced 5-methylcytosine levels. DNA methylation profiling identifies that this effect is specifically evident at CpG islands of critical trophoblast genes, such as *Gata3*, *Peg3*, *Socs3* and *Hand1*. As a consequence of the hypomethylated state, these factors are up-regulated in *Padi2/3* DKO TSCs, driving their premature differentiation. Our data uncover a critical epigenetic role for PADI2/3 in safeguarding the stem cell state of TSCs by modulating the DNA methylation landscape to restrict precocious trophoblast differentiation.

## 1. Introduction

The epigenetic code interprets the underlying genetic code to direct growth and development. It is comprised of modifications to the DNA itself, especially CpG methylation (“DNA methylation”), as well as various modifications to histone proteins. Amongst the best-studied histone modifications are methylation, acetylation, phosphorylation, ubiquitylation and poly(ADP)-ribosylation of amino-terminal amino acid residues, concentrated in particular on histones H3 and H4. Collectively, these modifications influence chromatin compaction and accessibility for transcription factors and transcriptional regulators, thereby directing which genes are switched on and off in any given cell. A much less well-studied modification is histone citrullination, despite the fact that this protein modification was identified 60 years ago [1]. Citrullination is the post-translational conversion of an arginine residue into the non-coded amino acid citrulline [2], which leads to the loss of a positive charge. Citrullination is carried out by a small family of enzymes called peptidylarginine deiminases (PADIs) [3]. Various histone proteins have been shown to be modified by citrullination, including histones H1, H2A, H2B, H3 and H4 [4,5,6].

In addition to causing the loss of a positive charge, histone citrullination eliminates the arginine residue as a target of methylation, a modification which has been tied to the positive regulation of stem cell pluripotency [7,8]. Moreover, the arginine residues that constitute citrullination targets commonly occur within the arginine–lysine–serine (“RKS”) motif, which has structural roles in chromatin dynamics [9]. Importantly, this motif occurs at histone H3-R8/K9/S10 and H3-R26/K27/S28. These residues are critical regulators of chromatin organization, transcriptional activity and mitotic competence. Amongst many other modifications that affect these particular amino acid residues is H3K9me3, an epigenetic repressive mark and hallmark of heterochromatin that also interacts with DNA methylation. This interaction is mediated by the bridging role of UHRF1 that couples H3K9me3 to DNA methyltransferase activity [10]. The citrullination of the arginine residue directly adjacent to H3K9me3 as well as to the Polycomb mark H3K27me3 can affect the binding capacity of various chromatin modifiers [11,12]. The consequences of histone citrullination are difficult to predict and are cell type-dependent because of these diverse facets of histone citrullination and its potential interactions and interferences with other histone marks and modifiers.

**Figure 1:**
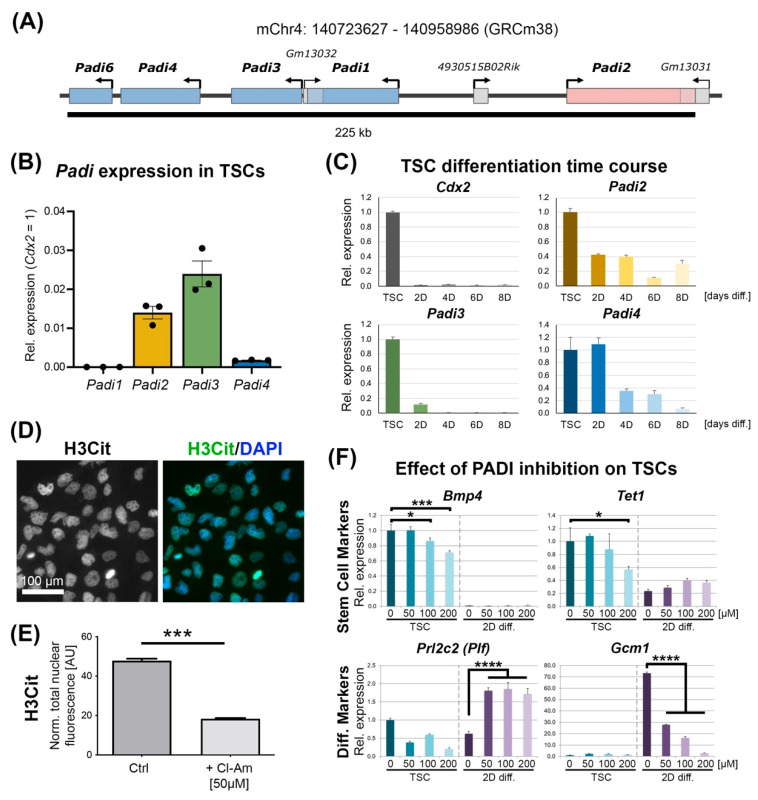
*Padi* expression in mouse trophoblast stem cells (TSCs). (**A**) Schematic representation of the *Padi* gene locus on mouse chromosome 4. The orientation of each gene is indicated by the arrow, and genes are colour-coded according to the direction with red genes being from left to right, and blue genes from right to left. (**B**) RT-qPCR analysis of *Padi1*–*Padi4* expression in TSCs. Data are shown relative to expression levels of the well-known TSC marker gene *Cdx2*, and are displayed as mean +/− S.E.M. (**C**) RT-qPCR analysis of *Padi2*, *Padi3* and *Padi4* expression dynamics during an 8-day (D) TSC differentiation time course. Data are expressed relative to TSC (i.e., stem) conditions and are displayed as mean +/− S.E.M. (*n* = 3). (**D**) Immunofluorescence staining against H3-R2/R8/R17 citrullination (H3Cit) in wild-type TSCs. (**E**) Quantification of H3-R2/R8/R17 citrullination (H3Cit) staining intensities in TSCs and in TSCs after 3 days of exposure to the pan-PADI inhibitor Chlor-Amidine (Cl-Am). Values are normalized to nuclear size. AU = arbitrary units. *** *p* < 0.001 (unpaired Student’s *t*-test). (**F**) RT-qPCR analysis of select stem cell and differentiation markers in TSCs grown under stem cell conditions (“TSCs”) and after 2 days of differentiation (“2D diff.”) in the presence of the indicated amounts of Chlor-Amidine. Data are expressed as relative to stem cell control (0 µM Cl-Am) and are displayed as mean +/− S.E.M. (*n* = 3 independent replicates). Statistical analysis was conducted for the relevant combinations of markers and conditions (TSC conditions for stem cell markers, 2D diff. conditions for differentiation markers) by 2-way ANOVA with Dunnett’s multiple comparisons test. * *p* < 0.05, *** *p* < 0.001, **** *p* < 0.0001.

The murine genome contains five *Padi* genes that are tightly clustered on mouse chromosome 4 and are believed to have arisen from gene duplication events (Figure 1A) [2]. *Padi6* is exclusively expressed in the oocyte and in the early preimplantation embryo [13]. The other four *Padi* members, *Padi1-4*, are more widely expressed with unique tissue distribution patterns and functional roles including in cellular differentiation, nerve growth, apoptosis, inflammation, gene regulation, and development [14].

The importance of histone citrullination in the stem cell context was highlighted by a study that positioned PADI4 as a central player of the pluripotency network in embryonic stem cells (ESCs) [15]. In ESCs, *Padi4* is the only PADI family member whose expression closely follows that of other pluripotency genes. High *Padi4* activity improves reprogramming efficiency and contributes to the maintenance of naïve pluripotency [15]. In contrast, in other non-stem cell contexts, PADI-mediated chromatin remodeling contributes to efficient cellular differentiation as has been shown, for example, for oligodendrocytes [4]. In the current study, we sought to investigate a potential role of PADI enzymes in trophoblast stem cells (TSCs). TSCs and ESCs can be regarded as developmental counterparts, as they are derived from the trophectoderm and from the inner cell mass of the blastocyst-stage mouse embryo, respectively. We found that TSCs most prominently express *Padi2* and *Padi3*, in contrast to ESCs. We demonstrate that these two PADIs function to safeguard the TSC state through a critical role in epigenome organization and specifically in the maintenance of DNA methylation. *Padi2/3* double knockout (DKO) TSCs display DNA hypomethylation at CpG islands associated with trophoblast giant cell-regulated genes, leading to their up-regulation. Thus, *Padi2* and *Padi3* are important modulators of the epigenomic landscape of trophoblast where they play critical roles in stem cell maintenance by preventing precocious terminal differentiation.

## 2. Material and Methods

### 2.1. TS Cell Culture

The wild-type blastocyst-derived TS-Rs26 TSC line (a kind gift of the Rossant lab, Toronto, ON, Canada) was cultured as described previously [16,17]. Briefly, TSCs were cultured under standard TSCs conditions: 20% foetal bovine serum (FBS) (Wisent Bioproducts 098150, Saint-Jean-Baptiste, QC, Canada), 1 mM sodium pyruvate (ThermoFisher Scientific 11360-039, Waltham, MA, USA), 1× anti-mycotic/antibiotic (ThermoFisher Scientific 15240-062, Waltham, MA, USA), 50 μM 2-mercaptoethanol (ThermoFisher Scientific (Gibco) 31350, Waltham, MA, USA), 37.5 ng/mL bFGF (Cambridge Stem Cell Institute, Cambridge, UK) and 1 μg/mL heparin in RPMI 1640 with L-Glutamine (ThermoFisher Scientific 21875-034, Waltham, MA, USA), with 70% of the medium pre-conditioned on mouse embryonic fibroblasts (CM). The medium was changed every two days, and cells passaged before reaching confluency. Trypsinization (0.25% Trypsin/EDTA) was carried out at 37 °C for about 5 min. Differentiation medium consisted of unconditioned TSC medium without bFGF and heparin.

For the generation of CRISPR/Cas9-mediated knockout TSCs, gRNAs that result in exon deletion and frameshift mutations in the remaining transcript sequence were designed using the http://crispor.tefor.net/interface (accessed between 1 January 2019 and 31 July 2021), chosen on the basis of high-specificity scores. gRNA sequences were cloned into the Cas9.2A.EGFP plasmid (Plasmid #48138 Addgene, Watertown, MA, USA) and sequence-verified. Transfection was carried out with Lipofectamine 2000 (ThermoFisher Scientific 11668019, Waltham, MA, USA) reagent according to the manufacturer’s protocol. EGFP-positive cells were single cell-sorted by the Flow Cytometry Core Facility at the University of Calgary into 96-well plates and expanded for KO TSC clone generation. Deletion of the targeted exon was confirmed by genotyping PCRs using primers spanning the deleted exon (expected product shorter than WT sequence), and with primers positioned within the deleted exon (negative control). EGFP-negative single cell-sorted cells from the same transfected TSC pools were used to generate WT control clones. Between 3 and 5 independent single cell-derived KO clones were generated and analyzed for each gene mutation alongside 8 single cell-derived WT clones.

### 2.2. RNA Isolation and RT-qPCR

Total RNA was extracted from cultured cells using TRI reagent (Sigma T9424, St. Louis, MO, USA) according to the manufacturer’s instructions. Between 1 and 2 µg of total RNA was used for cDNA synthesis with RevertAid H-Minus reverse transcriptase (Thermo Scientific EP0451, Waltham, MA, USA) and a mix of oligo-d(T)_18_ (ThermoFisher FERSO132, Waltham, MA, USA) and random primers (ThermoFisher FERSO142, Waltham, MA, USA). Quantitative (q)PCR was performed using the QuantiFast (Qiagen 25057, Hilden, Germany), QuantiNova (Qiagen 208057, Hilden, Germany) or SsoAdvanced Universal SYBR Green Supermix (BioRad 1725274, Hercules, CA, USA) and intron-spanning primer pairs on a QuantStudio 3 (ThermoFisher, Waltham, MA, USA) or CFX384 (Bio-Rad, Hercules, CA, USA) real-time PCR thermocycler. All primers were tested for efficiency and correct product amplification prior to use. In the few cases where primers were non-intron-spanning, RNA was DNAse treated before cDNA synthesis. The expression levels normalized to housekeeping gene *Sdha* are displayed as the mean relative to the WT control samples; error bars indicate the standard error of the means (S.E.M.) of at least three independent biological replicates. Where appropriate, Student’s *t*-test or ANOVA was performed to calculate the statistical significance of the expression differences (*p* < 0.05) using Graphpad Prism software.

### 2.3. Proliferation Analysis

TSCs seeded and grown at defined densities (100,000 cells/6-well plate) were washed and incubated for 10 min in 7 μM of fluorescence CellTrace CFSE reagent (ThermoFisher, Waltham, MA, USA) in phosphate-buffered saline (PBS), washed, and left to grow under standard TSC conditions for approximately 14 h. Then, the cells were trypsinized, centrifuged, and resuspended in PBS + 5% FBS. Excitation/emission spectra were obtained by flow cytometry, and proliferation profiles were determined by the Cell Tracking Wizard software (Flow Cytometry Core Facility, University of Calgary). Three clones per genotype (biological replicates) were assessed, and the proportions of cells in each generation were compared by F-test to determine the variance between the samples where f < 0.05 indicated unequal variances. Significant differences in the proportions of cells in each generation were determined by Student’s T-test (equal variances) or Welch’s T-test (unequal variances).

### 2.4. RNA Sequencing

Library construction for RNA sequencing was conducted with the NEB Ultra II Directional RNA Library Prep kit (E7760), and sequencing was performed on an Illumina NovaSeq 6000 (50 bp paired-end) by the University of Calgary’s Centre for Health Genomics and Informatics facility. All samples passed quality checks with fastqc and were then aligned to the mouse genome (GRCm38/mm10) using STAR [18]. Count tables were assembled with htseq-count using the reverse strand and intersection non-empty mode, along with all other default settings [19]. The visualization of sample variance and differential expression analysis was completed on R console (version 3.4.3 and 4.1.0) (https://www.R-project.org/) and using the SeqMonk software (https://www.bioinformatics.babraham.ac.uk/projects/seqmonk/) (accessed between 1 July 2019 and 31 July 2022). Prior to collapsing lanes, the intra-lane variability was assessed with centred-log ratio transformed scaled biplots. No lane replicates contained sufficiently high variance to be located in different PCA quadrants, and all lanes were then aggregated. Gene ontology analyses were performed with enrichR (https://maayanlab.cloud/Enrichr/), Panther (http://www.pantherdb.org/) and Metascape (https://metascape.org/) tools (accessed between 1 July 2019 and 30 June 2022).

### 2.5. DNA Methylation Profiling

MeDIP-seq was carried out as described previously [20,21]. Briefly, purified genomic DNA was sonicated to yield 150–600 bp fragments, and adaptors for paired-end sequencing ligated using NEB Next DNA Sample Prep Reagent Set 1 (New England Biolabs E7335S, E7645S, Ipswich, MA, USA). Immunoprecipitations were carried out using 1 µg DNA per sample, 2 μg anti-5 mC antibody (Eurogentec BI-MECY-0100, Seraing, Belgium), and 10 μL Dynabeads coupled with M-280 sheep anti-mouse IgG (ThermoFisher 112-01D, Waltham, MA, USA). Pulled-down DNA was used for library generation at UCalgary’s Centre for Health Genomics and Informatics facility. Indexed libraries were sequenced (50 bp paired-end) on an Illumina NovaSeq6000 sequencer. Raw fastq data were trimmed with trim-galore, using default parameters, and mapped to the Mus musculus GRCm38 genome assembly using Bowtie2 v2.2.6 (http://bowtie-bio.sourceforge.net/bowtie2/index.shtml) (accessed between 1 January 2022 and 31 March 2022), allowing only a single hit per read and guided by gene models from Ensembl v61. Data analysis was carried out using Seqmonk software (www.bioinformatics.babraham.ac.uk) (accessed between 1 July 2019 and 31 July 2022).

### 2.6. Immunofluorescence Staining

Cells were fixed with 4% paraformaldehyde (PFA) in PBS for 10 min and permeabilized with PBS, 0.1% Triton X-100 for 10 min. Blocking was carried out with PBS, 0.1% Tween-20, 0.5% BSA (PBT/BSA) for 30 min or overnight at 4 °C, followed by antibody incubation for 60 min. For 5-methylcytosine staining, the cells were permeabilized with PBS, 0.2% Triton X-100 overnight and DNA was denatured prior to staining with 2N HCl for 30 min at room temperature. Primary antibodies and dilutions (in PBT/BSA) were: H3R2/R8/R26Cit 1:300 (Abcam ab5103, Cambridge, UK); H3R8Cit 1:50–1:200 (Abcam ab219406, Cambridge, UK); H3R26Cit 1:50–1:200 (Abcam ab212082, Cambridge, UK); H4R3Cit 1:50–1:200 (Merck Millipore 07-596, Burlington, MA, USA); CDX2 1:250 (Biogenex MU392-UC, Fremont, CA, USA); SOX2 1:250 (R&D Systems AF2018, Minneapolis, MN, USA); gamma-Tubulin 1:100 (Abcam ab11316, Cambridge, UK); alpha-Tubulin 1:100 (NEB 2144S, Ipswich, MA, USA); H3K9me3 1:200 (Active Motif 61013, Carlsbad, CA, USA); 5-methylcytosine 1:250 (Epigentek A-1014-050, Farmingdale, NY, USA); and PEG3 1:300 (ThermoFisher PA599683, Waltham, MA, USA). Primary antibodies were detected with corresponding AlexaFluor488 or AlexaFluor568-conjugated secondary antibodies (1:500, ThermoFisher, Waltham, MA, USA), and nuclear counterstaining was performed with 4′,6-diamidino-2-phenylindole (DAPI).

### 2.7. Image Analysis

Images were taken with a Leica DMRE epifluorescence microscope using identical exposure and gain settings for staining intensity comparisons. Image analyses for nuclear size and fluorescence intensity measurements were performed with ImageJ software. All these analyses were performed in a blinded manner, with photographed areas selected purely in the DAPI channel before imaging the target-of-interest channel(s). For each staining, 5–7 images were taken for every TSC clone analyzed (*n* = 4 per genotype), with every image capturing approximately 30–100 cells. Thus, *n* ≥ 600 cells were analyzed for each genotype and every type of staining. Total nuclear fluorescence intensities were normalized to nuclear size. Statistical comparisons were performed in GraphPad Prism software using Student’s *t*-test or ANOVA as appropriate.

### 2.8. Primer Sequences

Primers used for RT-qPCRs and gRNAs for CRISPR targeting were as follows:
Padi1-FACAGACTACCCCCTCGGCAGPadi1-RCCACGGAAAGCCAGTCGGAGPadi2-FGCGCCCGTGGAACTCTACTCPadi2-RTGGCCATGAGCAGCCGAAATPadi3-FGGCAGTAGCGTTCTTCCCCGPadi3-RCCCCGTGCAGCATGTGGTATPadi4-FAAGCAGGGTTTTCGGCTGCTPadi4-RCGCCCGGTTCCAGTCGATAC9230102O04Rik-FGTTCATCGCACCCCAGAGGAC9230102O04Rik-RCCGCCTGGTCAATCCACAGTBmp4-FTGGCCCTCGACCAGGTTCATBmp4-RAAGGCTCAGAGAAGCTGCGGCdh15-FCCGAGTTCACCAAGGATGAGCdh15-RTCACGGCTCTCATAGTCCAGCdx2-FAGTGAGCTGGCTGCCACACTCdx2-RGCTGCTGCTGCTTCTTCTTGAEsrrb-FAGTACAAGCGACGGCTGGEsrrb-RCCTAGTAGATTCGAGACGATCTTAGTCAGata3_202-FCTTTGGTTCGGAAGTGCCCCGata3_202-RTGTCTGGGTGCTGACCGTTGGcm1-FACTTCTGGAGGCACGACGGAGcm1-RTCGGGATTTCAGCAGGAAGCGHand1-FTCTGCGCCTGGCTACCAGTTHand1-RTTTCGGGCTGCTGAGGCAACPrl2c2-FAACGCAGTCCGGAACGGGGPrl2c2-RTGTCTAGGCAGCTGATCATGCCAPrl3d1-FTTATCTTGGCCGCAGATGTGTPrl3d1-RGGAGTATGGATGGAAGCAGTATGACRnf144b-FGCGCCCAGATGATGTGCAAGARnf144b-RCCCCACTACCTGTGTTCGGTSdha-FTGGTGAGAACAAGAAGGCATCASdha-RCGCCTACAACCACAGCATCASynb-FTCCGGAAAGGGACCTGCCCASynb-RCAGCAGTAGTGCGGGGTGCCTet1-FGAGCCTGTTCCTCGATGTGGTet1-RCAAACCCACCTGAGGCTGTTTpbpa-FACTGGAGTGCCCAGCACAGCTpbpa-RGCAGTTCAGCATCCAACTGCGPadi2 gRNA1CATGGTTGAGTATCTTGTGTPadi2 gRNA2GCCTGAGCCACAGAAACAGTPadi3 gRNA1AGCTCAACATCTCTTAGCCAPadi3 gRNA2GTCTGTGTAAAAGAAGCAGTPadi4 gRNA1GAGTACAATTCAAAGGCCAGPadi4 gRNA2GTCAATACTTCAGTGCCACT

## 3. Results

### 3.1. Padi Expression in TSCs

To establish which *Padi* members are expressed in TSCs, we started our analysis by determining the transcript levels of *Padi1–4* by RT-qPCR. We did not include *Padi6* in this analysis because of its highly restricted expression in oocytes and 1-cell embryos only; the confirmation of the lack of *Padi6* in TSCs was further corroborated by our RNA-seq data generated as part of this project (see below). *Padi2*, *Padi3* and *Padi4* were readily detected in TSCs, with *Padi3* exhibiting the highest expression, followed by *Padi2* (Figure 1B). *Padi3* transcript levels reached approximately 2–3% of that of the well-known and highly expressed TSC marker *Cdx2* (Figure 1B), which is similar in abundance to other important epigenetic regulators such as *Tet1* [22]. In contrast, we were unable to detect *Padi1* mRNA at any appreciable level with multiple primer pairs (Figure 1B).

TSCs can be induced to differentiate into the various trophoblast cell types of the placenta when vital growth factors are withdrawn from the TSC culture medium, notably FGF4 as well as a TGFb component contained in mouse embryonic fibroblast-conditioned medium (CM) [16,23,24]. When assessed across a differentiation time course, it became obvious that all TSC-expressed *Padi*’s were most abundant under stem cell conditions and down-regulated upon differentiation (Figure 1C). This was most acutely evident for *Padi3* whose expression was strictly associated with the stem cell state of trophoblast, akin to the stem cell marker *Cdx2*.

We then examined TSCs for the presence of PADI-conferred histone citrulline modifications, and detected reliable, nuclear staining with the antibody against histone H3R2/R8/R17 (Figure 1D and Figure S1A). We also tested antibodies against several other histone citrulline marks including H3R8Cit, H3R26Cit and H4R3Cit, but failed to detect any immunoreactivity (data not shown). This may be due to the reduced abundance of the respective modifications and/or due to poorer antibody specificity. The staining intensity of H3R2/R8/R17 was significantly decreased upon treatment of TSCs with Chlor-Amidine, a pan-PADI inhibitor (Figure 1E). Short-term treatment of TSCs with Chor-Amidine for 2 days resulted in a dose-dependent down-regulation of acutely sensitive stem cell markers such as *Bmp4*, *Tet1, Esrrb* and *Cdx2* (Figure 1F and Figure S1B) [22,25]. In the absence of FGF4 and CM, the exposure of TSCs to Chlor-Amidine caused a down-regulation of the early differentiation markers *Rnf144b* and *Cdh15* but an up-regulation of the trophoblast giant cell (TGC) marker *Prl2c2* (=*Plf*, Proliferin), indicative of an accelerated TGC differentiation rate (Figure 1F and Figure S1C). At the same time, the syncytiotrophoblast precursor marker *Gcm1* was drastically down-regulated (Figure 1F).

Overall, these data established that TSCs express a specific subset of *Padi* family members, and that their activity is associated with the stem cell state of trophoblast.

### 3.2. Padi KO Reduces the Transcriptional Heterogeneity of TSCs

**Figure 2:**
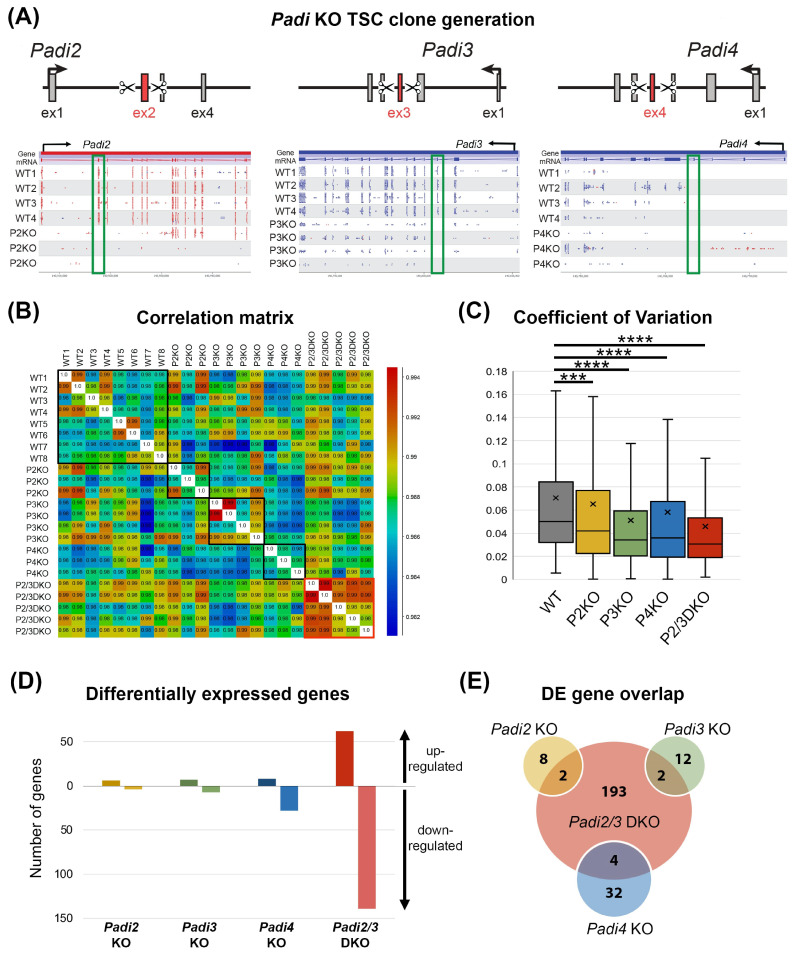
*Padi* knockout (KO) TSC generation and transcriptomic analysis. (**A**) Depiction of the *Padi* genes with the exons targeted for deletion by CRISPR/Cas9-mediated knockout highlighted in red. Deletion of the targeted exons results in frameshift mutations and premature translational termination. SeqMonk screenshots of RNA-seq data of wild-type (WT) and independently derived *Padi2* (P2)-, *Padi3* (P3) and *Padi4* (P4)-KO clones across the relevant genomic regions are shown underneath, with the targeted exons highlighted by the green rectangle. A complete absence of reads across the deleted exons provides an independent confirmation of correct gene ablation. Of note, *Padi4* exon 4 expression was detectable at low levels in some WT TSCs. (**B**) Correlation matrix of global gene expression profiles across all TSC clones of the indicated genotypes. Rectangles highlight the relevant samples comparing inter-clone variability in each genotype. *Padi2/3* double KO (DKO) clones are the most similar to each other, whereas WT clones display a higher extent of clonal variability. (**C**) The coefficient of variation of the top 10,000 expressed genes in WT and *Padi* KO/DKO clones. All *Padi* KOs exhibit reduced transcriptional heterogeneity. This effect is most pronounced in the *Padi2/3* DKO TSCs. Statistical analysis was conducted by one-way ANOVA with Dunnett’s multiple comparisons test. *** *p* < 0.001, **** *p* < 0.0001. (**D**) Diagram of up- and down-regulated genes in the various KO/DKO TSC clones as determined by DESeq2 analysis (*p* < 0.05). (**E**) Overlap of differentially expressed (DE) genes in the various *Padi* KO/DKO TSCs.

Since chemical inhibitors do not affect each PADI with the same specificity and efficacy, they cannot be used to unambiguously tease out the role of individual *Padi* genes. To overcome this problem, we generated CRISPR/Cas9-mediated knockout (KO) TSCs for each of the TSC-expressed *Padi* genes, namely *Padi2*, *Padi3* and *Padi4* (Figure 2A). We also established *Padi2/3* double KO (DKO) TSC clones which simultaneously lacked both of the most abundantly expressed PADIs. For each of these mutations, we generated ≥3 independently derived KO/DKO TSC clones.

Our first assessment strategy was to perform transcriptional profiling by RNA-seq of (D)KO and wild-type (WT) control clones to identify the overarching effects of *Padi* deficiency on TSCs. Globally, the samples clustered by differentiation state (stem cell state or 2 days of differentiation) irrespective of genotype (Figure S2A). Because of the expression dynamics of *Padi*’s during differentiation (Figure 1C), we focused the subsequent analysis on the data obtained from TSC conditions. When comparing the overall sample similarity of these datasets more closely, it was evident that the *Padi2/3* DKO TSC clones were highly similar to each other, whereas the individual WT clones showed a wider range of variability despite the fact that they were isolated in parallel by the same single cell-sorting and expansion procedure (Figure 2B). To examine this observation more closely, we calculated the coefficient of variation (CoV) on the top 10,000 expressed genes and found that all *Padi* KO clones had a significantly lower CoV than WT clones (Figure 2C). This reduction in transcriptional heterogeneity was most pronounced in the *Padi2/3* DKO cells, and second lowest in *Padi3* KO clones, in line with *Padi3* being the most highly expressed family member.

The differential gene expression analysis by DESeq2 identified only very few dysregulated genes in the single KOs (Figure 2D). Interestingly, *Padi4* KO TSCs exhibited a larger number of differentially expressed (DE) genes than *Padi2* KO and *Padi3* KO TSCs (Figure 2D,E), despite the comparatively low expression levels of *Padi4* in TSCs. This finding may be due to the fact that PADI4 contains a canonical nuclear localization signal and, accordingly, has a prominent functional role in the nucleus [26]. Overall, however, a much more robust number of 201 DE genes was identified between WT and *Padi2/3* DKO TSCs (Figure 2D). Of these DE genes, the majority were down-regulated in *Padi2/3* DKO compared to WT cells (139/201 = 69%). Interestingly, the down-regulated genes were enriched for pathways that are of critical importance for TSC maintenance, notably TGFb and FGF signaling (Figure S2B). Due to the small number of DE genes identified in the single KOs, the over-lap with de-regulated genes in DKO cells was minimal (Figure 2E). Given that the *Padi2/3* DKO TSCs exhibited by far the most profound effects on their transcriptomes, we focused the subsequent study on the analysis of these double mutant cells specifically.

### 3.3. Padi2/3 DKO TSCs Are Prone to Differentiate into Large Trophoblast Giant Cells

**Figure 3:**
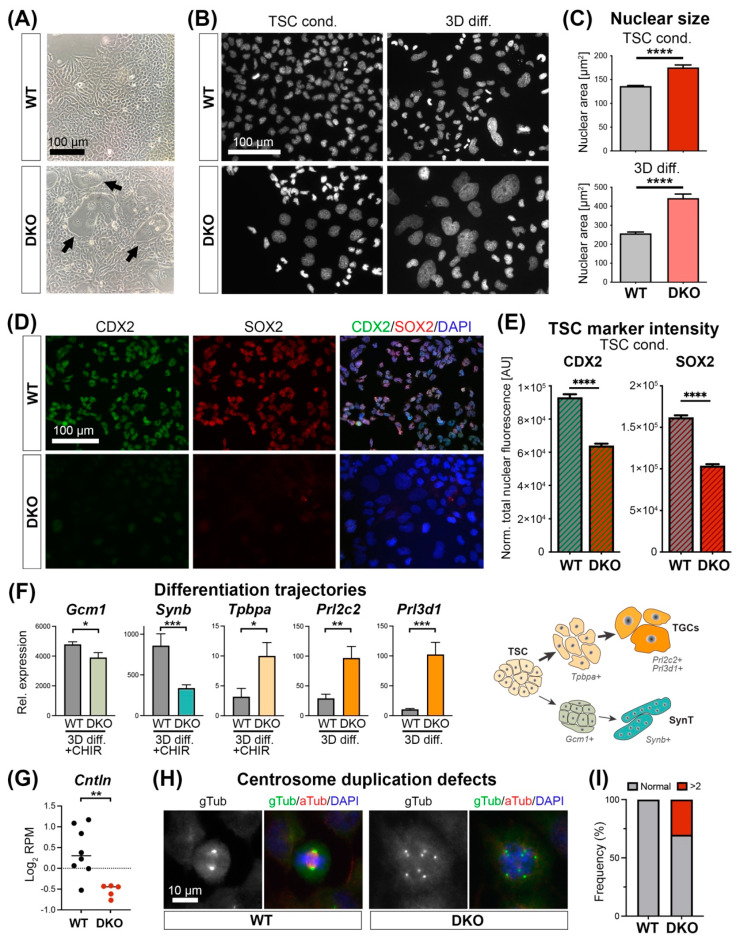
*Padi2/3* DKO TSCs exhibit diminished stem cell potential and are prone to differentiate into large trophoblast giant cells (TGCs). (**A**) Phase contrast images of wild-type (WT) and *Padi2/3* DKO TSCs. Arrows point to extremely large trophoblast giant cell-like cells in the DKO. (**B**) DAPI-stained nuclei of WT and *Padi2/3* DKO TSCs grown under stem cell conditions (TSC cond.) and after 3 days of differentiation (3D diff). Note the extremely large nuclei in the DKO cells even under TSC conditions. (**C**) Independent and unbiased quantification of nuclear sizes in WT and *Padi2/3* DKO cells. Statistical analysis by unpaired Student’s *t*-test (**** *p* < 0.0001; *n* = 4 independent clones per genotype with >150 cells assessed each). (**D**) Immunofluorescence staining on WT and *Padi2/3* DKO TSCs for trophoblast stem cell markers CDX2 and SOX2. (**E**) Quantification of fluorescence intensities of CDX2 and SOX2 staining. AU = arbitrary units. Statistical analysis by unpaired Student’s *t*-test (** *p* < 0.01, **** *p* < 0.0001; *n* = 4 independent clones per genotype with >150 cells assessed each). (**F**) RT-qPCRs for relevant trophoblast differentiation markers on WT and *Padi2/3* DKO TSC after 3 days of standard differentiation or 3 days of differentiation in the presence of CHIR99021 (CHIR), which promotes differentiation specifically into syncytiotrophoblast layer-II cells (SynT). The main differentiation trajectories and corresponding marker genes are depicted. *Padi2/3* DKO TSCs are prone towards TGC differentiation and significantly inhibited towards SynT differentiation even when pushed towards that lineage with CHIR. Values are displayed relative to TSC (i.e., stem) conditions. Statistical analysis by unpaired Student’s *t*-test (* *p* < 0.05; ** *p* < 0.01, *** *p* < 0.001; *n* = 4 independent clones per genotype). (**G**) Expression levels of *Cntln* in WT and *Padi2/3* DKO TSCs as determined by RNA-seq. Statistical analysis by unpaired Student’s *t*-test (** *p* < 0.01). (**H**) Immunostaining for alpha- and gamma-tubulin demarcating spindle fibres and centrosomes, respectively. (**I**) Quantification of the number of mitotic cells exhibiting 2 (“Normal”) or more than 2 centrosomes in WT and *Padi2/3* DKO TSCs. Data are from 65 WT and 43 *Padi2/3* DKO cells that displayed chromosome condensation indicative of (pro)metaphase by DAPI staining.

Routine culture of *Padi2/3* DKO cells under stem cell conditions quickly revealed a phenotypic difference. Thus, it was obvious that they contained some very large cells that showed all typical morphological characteristics of TGCs (Figure 3A), a finding that was never observed in any of the WT clones. Systematic measurements and analyses of nuclear size distribution demonstrated an increase in the largest ranges (≥400 µm^2^) in DKO cells, with similar albeit less pronounced trends in evidence even in the single *Padi* KOs (Figure S3A). In *Padi2/3* DKO stem cell cultures, these cells were already as large as, if not larger than, TGCs that emerged in WT cultures after 3 days of differentiation (Figure 3B,C). By this time, some DKO cells had become truly gigantic (Figure 3B). As perhaps expected with a fraction of cells exhibiting such precocious differentiation even under stem cell conditions, *Padi2/3* DKO cells displayed a decrease in proliferation rates on a population-wide scale (Figure S3B).

The precocious TGC differentiation phenotype together with the down-regulation of genes related to TGFb and FGF signaling suggested that *Padi2/3* DKO TSCs exhibited a reduced capacity to maintain the stem cell state. To investigate this further, we performed immunostaining for the stem cell transcription factors CDX2 and SOX2 followed by unbiased quantification of corrected total fluorescence intensities (Figure 3D). This analysis demonstrated a significant reduction in the abundance of CDX2 and SOX2, both critical components of the TSC self-renewal network (Figure 3E) [27,28,29]. We then assessed whether this reduction in stem cell factors was associated with a general differentiation-promoting phenotype, or whether *Padi2/3* deficiency triggered differentiation along a particular trajectory, namely towards TGC or syncytiotrophoblast (SynT) formation (Figure 3F). For this purpose, we differentiated WT and DKO TSCs for 3 days in the presence or absence of CHIR99021 (CHIR), which drives TSC differentiation specifically into SynT-II cells [30]. Remarkably, even when forced towards SynT formation by CHIR, *Padi2/3* DKO cells were highly refractory towards differentiation into this lineage; instead, they preferentially differentiated into the TGC lineage (Figure 3F).

Prompted by this finding and by the visual appearance of extremely large TGCs, our attention was drawn to the pronounced down-regulation of a particular gene, *Cntln*, which was evident in the RNA-seq data (Figure 3G). *Cntln* encodes for Centlein, a centriole adhesion and microtubule stabilization factor. We previously reported that centrosome duplication defects due to *Tet1* deficiency cause TSCs to undergo precocious differentiation into TGCs [22]. Therefore, we co-stained WT and *Padi2/3* DKO TSCs for gamma-Tubulin as a marker of centrosomes, and for alpha-Tubulin as a marker of spindle fibres. Mitotic WT cells always contained two centrosomes oriented towards opposing spindle poles. In stark contrast, 30% of *Padi2/3* DKO cells with condensed chromosomes contained more than two centrosomes (Figure 3H,I). This cell biological defect may underlie the appearance of prematurely differentiating, over-enlarged TGCs.

### 3.4. Padi2/3 Deficiency Causes DNA Methylation Defects

**Figure 4:**
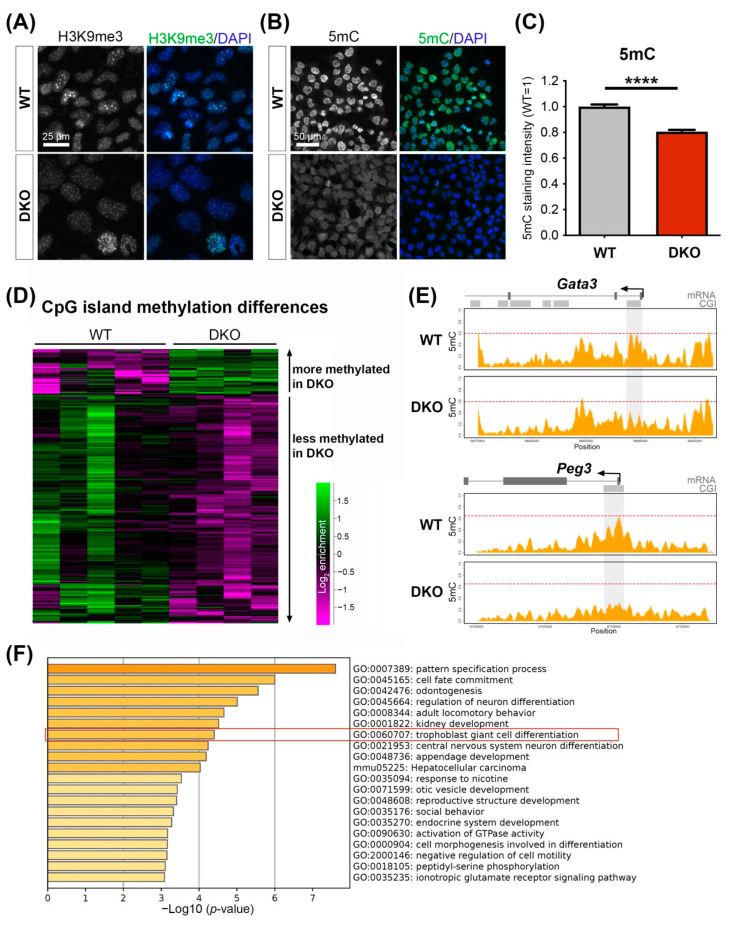
*Padi2/3* DKO TSCs exhibit an altered epigenomic landscape. (**A**) Immunofluorescence staining for H3K9me3 on WT and *Padi2/3* DKO TSCs reveals fewer bright heterochromatic foci (chromocentres) and instead, an overall reduced and more finely dispersed staining pattern of this heterochromatic mark in *Padi2/3* DKO cells. (**B**) 5-methylcytosine (5mC) immunostaining on WT and *Padi2/3* DKO TSCs. (**C**) Quantification of 5mC fluorescence intensities shows reduced amounts of 5mC in DKO cells. Statistical analysis by unpaired Student’s *t*-test (**** *p* < 0.0001; *n* = 4 independent clones per genotype with >150 cells assessed each). (**D**) Heatmap of read count enrichment across developmentally regulated CpG islands (CGIs) [32] as determined by meDIP-seq profiling. (**E**) Wiggle plots of 5mC enrichment across the *Gata3* and *Peg3* loci. Gene structure (exons) and CGIs are displayed on top of the graphs. The differentially methylated CGIs are shaded. The red dotted line demarcates the level of maximal enrichment in WT cells, and is drawn at corresponding levels in the DKO graph to help visualize the reduction in 5mC enrichment. (**F**) Metascape gene ontology analysis showing significantly enriched terms for genes associated with hypomethylated CGIs in *Padi2/3* DKO TSCs.

Since one of the main histone citrullination sites falls into the critical H3-R8/K9/S10 domain, we asked whether the level or distribution of the heterochromatin-associated modification H3K9me3 is altered in *Padi2/3* DKO TSCs. Indeed, staining against this well-established repressive histone mark appeared globally weaker (Figure 4A). Most notably, *Padi2/3* DKO cells did not contain as many brightly stained coalescent chromocentric foci of pericentric heterochromatin; instead, they exhibited a more finely grained punctate staining pattern with far fewer heterochromatic foci compared to WT TSCs (Figure 4A). The repressive H3K9me3 modification is tightly linked to DNA methylation. These two marks often form redundant epigenetic repressive layers whose distribution is mechanistically coupled through the function of UHRF1 [10]. Indeed, immunofluorescent detection of 5-methylcytosine (5mC) showed a significantly lower nuclear staining intensity in *Padi2/3* DKO compared to WT TSCs (Figure 4B,C).

This apparent reduction in 5mC levels prompted us to investigate the genome-wide distribution of DNA methylation by meDIP-seq [20,31]. This analysis was aimed at identifying the genomic regions of regulatory significance that cannot be resolved by histological staining techniques. Globally, the DNA methylation profiles were very similar between WT and *Padi2/3* DKO TSCs across the uniquely mappable genome, as expected (Figure S4A). However, despite global similarity, we previously found that DNA methylation differences at developmentally regulated CpG islands (CGIs) consistently provided the most informative readout of functionally meaningful DNA methylation differences [20,21,32]. When assessing enrichment at these genomic features, we identified 373 differentially methylated CGIs between WT and *Padi2/3* DKO TSCs. In line with the immunofluorescence staining results, the vast majority (311/373 = 83%) of these CGIs were hypomethylated in the *Padi2/3* DKO cells (Figure 4D). This included promoter-spanning CGIs at important trophoblast genes such as *Gata3*, *Peg3* and *Socs3* (Figure 4E, Figures S4B and S5), as well as a CGI in the 3′-region of the *Hand1* gene (Figure S4B). Notably, gene ontology analysis on genes associated with hypomethylated CGIs (within 2kb) produced the term “trophoblast giant cell differentiation” (Figure 4F), a highly significant hit as placenta-associated terms are often under-represented in such databases.

Thus, our data demonstrated that *Padi2/3* DKO TSCs exhibit defects in the normal abundance and distribution of heterochromatin marks. In particular, they harbour reduced levels of DNA methylation at CGIs of important trophoblast genes, which may be caused by the reduced expression levels of the DNA methyltransferase cofactor gene *Dnmt3l* (Figure S4C).

### 3.5. DNA Hypomethylation Licenses Trophoblast Genes for Precocious Up-Regulation

**Figure 5:**
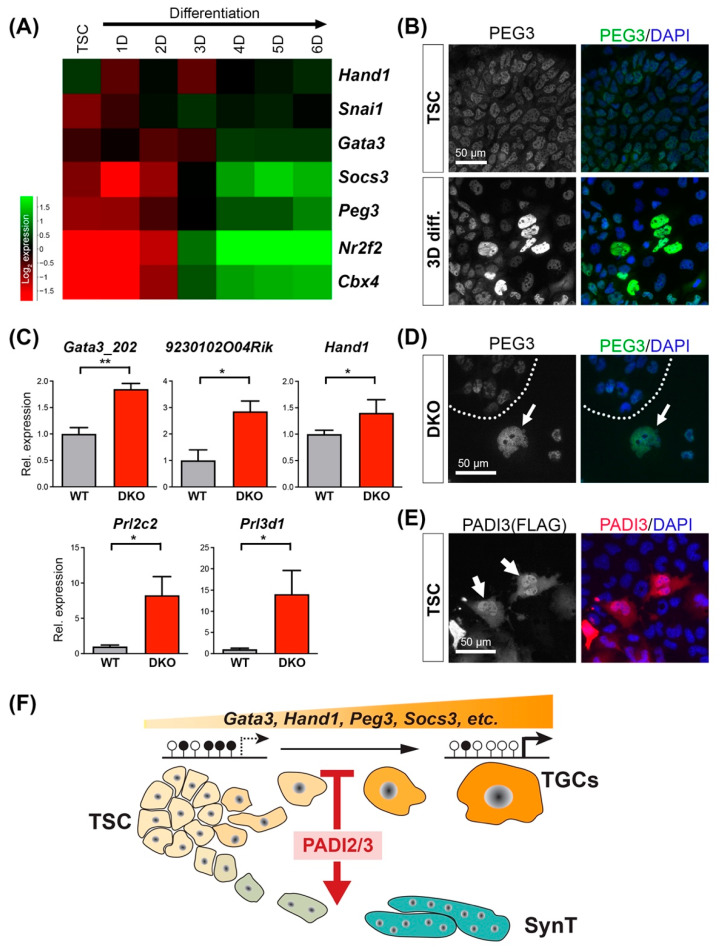
CGI hypomethylation licenses trophoblast differentiation-promoting genes for precocious up-regulation. (**A**) Expression dynamics of trophoblast differentiation-relevant genes that are associated with hypomethylated CGIs in *Padi2/3* DKO cells across a TSC differentiation time course. (**B**) Immunofluorescence staining for PEG3 indicates its up-regulation in TSCs after 3 days of differentiation. (**C**) RT-qPCR analysis showing the up-regulation of genes associated with hypomethylated CGIs (*Gata3_202*, *9230102O04Rik*, *Hand1*) and TGC differentiation markers (*Prl2c2*, *Prl3d1*) in *Padi2/3* DKO TSCs under stem cell conditions. Data are expressed relative to WT TSCs and are displayed as the mean +/− S.E.M. Statistical analysis by unpaired Student’s *t*-test (* *p* < 0.05; ** *p* < 0.01; *n* = 4 independent clones per genotype). (**D**) Immunofluorescence staining for PEG3 showing stronger expression in the enlarged TGCs (arrow) that are evident in *Padid2/3* DKO TSCs under stem cell conditions. (**E**) Immunostaining for FLAG-tagged PADI3 in TSCs revealing unequivocally nuclear localization (arrows) in TSCs. (**F**) Model depicting the role of PADI2/3 in TSCs. Both PADIs act synergistically to prevent precocious TGC differentiation by maintaining DNA methylation at CGIs that are associated with key trophoblast genes, preventing their premature up-regulation. This inhibitory effect towards TGC formation allows for the sufficient differentiation of SynT cells.

When assessing the transcriptional dynamics of genes associated with hypomethylated CGIs, we found that around half of them were up-regulated during normal TSC differentiation (Figure S6A). This included the transcription factors and chromatin regulators *Cbx4*, *Gata3*, *Hand1*, *Nr2f2* (Coup-tfII), *Peg3*, the cytokine signaling component *Socs3*, and the EMT marker *Snai1* (Figure 5A,B). These data are in line with previous reports on the expression profiles of many of these genes [33,34,35,36,37]. It is also noteworthy that all of these genes were already expressed at low levels in TSCs but increased in abundance during the differentiation process towards TGCs.

Since our data showed that *Padi2/3* DKO TSCs are prone to precociously differentiate into TGCs, we hence expected these genes to be up-regulated in DKO cells, specifically in the subset that undergoes premature differentiation (Figure 3). This was indeed the case. Thus, we found higher expression levels for two transcripts regulated by the differentially methylated *Gata3* CGI, specifically the transcript isoform *Gata3_202* and the overlapping transcript *9230102O04Rik*, in bulk TSC samples of *Padi2/3* DKO clones (Figure 5C). We also detected elevated transcript levels of *Hand1* (Figure 5C), alongside an up-regulation of classical TGC markers such as Proliferin (*Prl2c2*) and placental lactogen 1 (*Prl3d1*) in *Padi2/3* DKO TSCs. For other CGI-associated factors such as *Peg3*, we observed markedly higher immunostaining signals in the morphologically obvious TGCs that appear in *Padi2/3* DKO clones even under stem cell conditions (Figure 5D and Figure S6B). These data show that DNA hypomethylation at critical, dynamically regulated trophoblast genes creates a permissive state that allows these factors to become precociously up-regulated in *Padi2/3* DKO cells.

### 3.6. A Novel Nuclear Role for PADI3

Our data imply a nuclear function in TSCs for both, PADI2 and PADI3. Of the PADI gene family, only PADI4 contains a canonical nuclear localization signal [26,38]. However, subcellular localization to the nucleus has also been shown for PADI2, which is in line with the identification of histones as direct PADI2-mediated citrullination targets, specifically at sites H3R26 and H3R2/R8/R17 [4,39,40,41]. For PADI3, a nuclear role has not been reported to date. Therefore, we tested the possibility that a fraction of PADI3 may also locate to the nucleus where it could directly affect histones and/or other nuclear proteins. To obtain unambiguous immunolocalization results, we generated a FLAG-tagged *Padi3* expression construct that was transfected into TSCs, in parallel with a control plasmid encoding a FLAG-tagged version of the transcription factor *Elf5* [42]. Anti-FLAG staining revealed a predominantly nuclear localization of ELF5, as expected (Figure S6C). Notably, for PADI3, staining was detected in both the cytoplasm and the nucleus. Thus, we identified a significant number of TSCs in which a fraction of PADI3 unambiguously located to the nucleus and was even enriched in this compartment (Figure 5E). These data demonstrate a novel nuclear localization and role for PADI3.

## 4. Discussion

The PADI enzymes have received considerable attention over recent years for their role as epigenetic modifiers. Such a function has been highlighted in particular by a report that *Padi4* is a key pluripotency factor in the self-renewal circuit of ESCs, a role exerted by the citrullination of histone H1 leading to chromatin decondensation [15]. In addition, H3R26Cit has also been shown to contribute to the maintenance of ESC pluripotency by preferential binding to SMARCAD1, which in turn suppresses heterochromatin formation [12]. In non-ESC contexts, by contrast, PADI-mediated epigenome remodeling is required for proper differentiation, as—for example—in oligodendrocyte lineage progression and myelination [4]. PADIs are also important for the normal progression of preimplantation development, a function that has been tied in particular to PADI1 [43]. Collectively, these data demonstrate that PADIs play essential roles in stem cell biology and development. For this reason, we were interested in exploring a potential function of PADIs in stem cells of the trophoblast compartment.

In the current study, we show that TSC maintenance critically relies on PADI expression. However, unlike in ESCs where *Padi4* is the dominant family member, TSCs preferentially express *Padi3* and *Padi2*, while *Padi4* expression is modest and *Padi1* virtually absent. Accordingly, although we detect some subtle functional changes in *Padi2*-, *Padi3*- and *Padi4*-single KO TSCs, the most pronounced phenotypic and transcriptional alterations are evident in *Padi2/3* DKO TSCs. This effect does not appear to be simply additive, but rather points to synergistic consequences of *Padi2/3* compound deficiency, suggesting that both PADIs have partially redundant functions in convergent pathways. Our finding of a nuclear localization of PADI3, together with the established role of PADI2 in this subcellular compartment [40], further underscores this point.

The most notable morphological phenotype of *Padi2/3*-mutant TSCs is the appearance of extremely large TGCs even under stem cell conditions. The size of these nuclei is at least equivalent to, if not larger than, that of WT TSCs after 3 days of differentiation, suggesting that these cells undergo excessive rounds of endoreduplication. This phenotype may be caused by the centrosome duplication defects that are apparent in a sizeable fraction of metaphase *Padi2/3* DKO TSCs and that are highly reminiscent of the mitotic defects we previously observed in *Tet1* KO and *Tet1/2* DKO TSCs [22]. Due to the endoreduplicative capacity of trophoblast cells, the presence of superfluous centrosomes results in TGC differentiation as an ‘escape’ pathway in this cell lineage, whereas it would cause apoptosis in most other cell types.

Coincident with the increased TGC differentiation propensity of *Padi2/3* DKO TSCs is a decrease in stem cell marker expression, such as for transcription factors CDX2 and SOX2 [25,28,29]. However, syncytiotrophoblast formation is dramatically blocked in *Padi2/3* DKO TSCs and upon pan-PADI inhibitor treatment, demonstrating that these cells are not just simply more prone to differentiate, but are restricted in their differentiative capacity towards the TGC lineage. This differentiation bias may be explained by the influence of PADIs on WNT signaling, a critical pathway for syncytiotrophoblast differentiation [44]. Intriguingly, this loss of plasticity is reflected by the reduced transcriptional heterogeneity that we observe in all KOs, but most significantly in the *Padi2/3* DKO. Thus, although *Padi2/3* deficiency does not entirely preclude the maintenance of mutant TSC cell lines, it lowers their stem cell state, reduces their differentiative plasticity and makes them more prone to differentiate specifically into TGCs (Figure 5F). Therefore, our data demonstrate a critical role of PADI2 and PADI3 in maintaining the full differentiative plasticity and self-renewal potential of TSCs, which is akin to PADI function in ESCs.

These phenotypes of *Padi2/3* DKO TSCs are accompanied by marked changes in the epigenomic architecture. Thus, we show that *Padi2/3* double-mutant TSCs fail to form the typical large, H3K9me3-marked and DAPI-bright chromocenters and instead contain smaller, more dispersed H3K9me3-positive heterochromatic foci. This altered distribution of a key repressive histone mark may be caused by steric interactions between the adjacent R8 and K9 residues on histone H3. Moreover, these changes may be affected by heterochromatin protein 1 (HP1), the major H3K9me3 binding protein, being a citrullination target itself [45]. Perhaps even more notable is the impact of *Padi2/3* deletion on the DNA methylation landscape. Our data demonstrate a role of PADI2/3 in maintaining moderate DNA methylation levels at CGIs of multiple well-known trophoblast genes, including *Gata3*, *Peg3*, *Hand1* and *Socs3*. In the absence of PADI2/3, the hypomethylated state licenses these genes to become up-regulated (Figure 5F). Since elevated expression of these factors is known to drive TSC differentiation [33,36,46,47], the loss of methylation can directly explain the premature differentiation phenotype of *Padi2/3* DKO TSCs. These data also align with our earlier observations that the demethylation of TSCs induces TGC differentiation [48].

Multiple mechanisms can be envisaged of how PADI2/3 may affect the DNA methylation state. It is known that DNA methyltransferases are stabilized by citrullination, which leads to increased DNA methylation levels, an effect that has been specifically shown for DNMT3A [49,50]. Due to the mutually reinforcing interactions between H3K9me3 and DNA methylation, it is also possible that the reduction of H3K9me3 induces the hypomethylated state [51]. Moreover, we find that *Padi2/3* DKO TSCs express significantly less *Dnmt3l,* which may contribute to the loss of DNA methylation and epigenetic repressive assembly complexes even in non-gametic cells [52,53]. In any case, our demonstration that a fraction of PADI3 protein is localized to the nucleus reveals a novel subcellular compartment of PADI3 function and suggests partially overlapping targets between PADI2 and PADI3.

Taken together, in this study, we identify a novel role for PADI2/3 in safeguarding the stem cell state of TSCs by epigenetically restricting precocious differentiation through the maintenance of DNA methylation at critical trophoblast genes.

## Data Availability

Sequence data were deposited under accession GSE208305.

## Supplementary Materials

The following supporting information can be downloaded at: https://www.mdpi.com/article/10.3390/cells11162466/s1, Figure S1: Effect of PADI inhibition on TSC differentiation, Figure S2: Consequences of PADI deletion on TSC transcriptomes, Figure S3: Padi2/3 ablation causes TSCs to become larger and less proliferative, Figure S4: Padi2/3 DKO TSCs exhibit reduced DNA methylation levels at key trophoblast gene loci, Figure S5: DNA methylation differences are comparable between independently derived cell clones, Figure S6: Regulation of genes associated with hypomethylated CGIs.
