# Supplementary material for: Padi2/3 Deficiency Alters the Epigenomic Landscape and Causes Premature Differentiation of Mouse Trophoblast Stem Cells
Source: Cells. 2022 Aug 9;11(16):2466. doi: 10.3390/cells11162466 (PMC9406452; doi:10.3390/cells11162466)
Supplement: Supplementary file 1 [file cells-11-02466-s001.zip › cells-1843514 Supplemental Material.pdf]

Padi2/3 Deficiency Alters the Epigenomic Landscape and Causes Premature Differentiation of Mouse Trophoblast Stem Cells

Noura N. Ballasy[1+], Elizabeth A. Bering[1+], Caroline Kokorudz[1], Bethany N. Radford[1], Xiang Zhao[2], Wendy Dean[2,3*] and Myriam Hemberger[1,3*]

Supplemental Material containing:

- Main Figures 1-5 (full size)
- Supplementary Figures S1 - S6 including legends

Figure 1

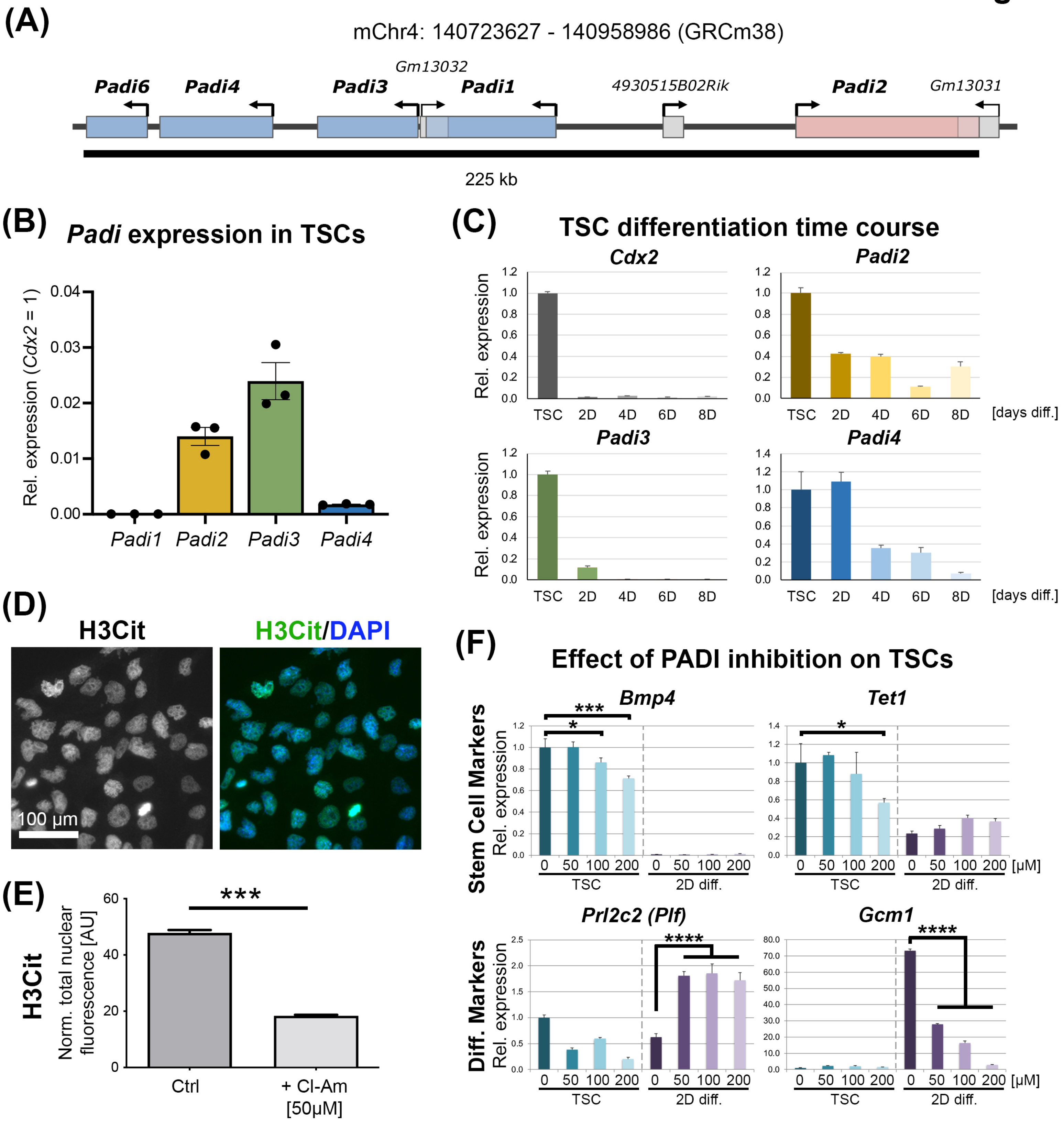
(A)
mChr4: 140723627 - 140958986 (GRCm38)
Padi6
Padi4
Padi3
Gm13032
Padi1
4930515B02Rik
Padi2
Gm13031
225 kb
(B)
Padi expression in TSCs
Rel. expression (Cdx2 = 1)
0.04
0.03
0.02
0.01
0.00
Padi1
Padi2
Padi3
Padi4
(C)
TSC differentiation time course
Cdx2
Padi2
Padi3
Padi4
Rel. expression
TSC
2D
4D
6D
8D
[days diff.]
(D)
H3Cit
H3Cit/DAPI
100 µm
(E)
H3Cit
Norm. total nuclear fluorescence [AU]
60
40
20
0
***
Ctrl
+ Cl-Am [50µM]
(F)
Effect of PADI inhibition on TSCs
Stem Cell Markers
Bmp4
Tet1
Diff. Markers
Prl2c2 (Plf)
Gcm1
Rel. expression
***
*
****
0
50
100
200
[µM]
TSC
2D diff.

Figure 2

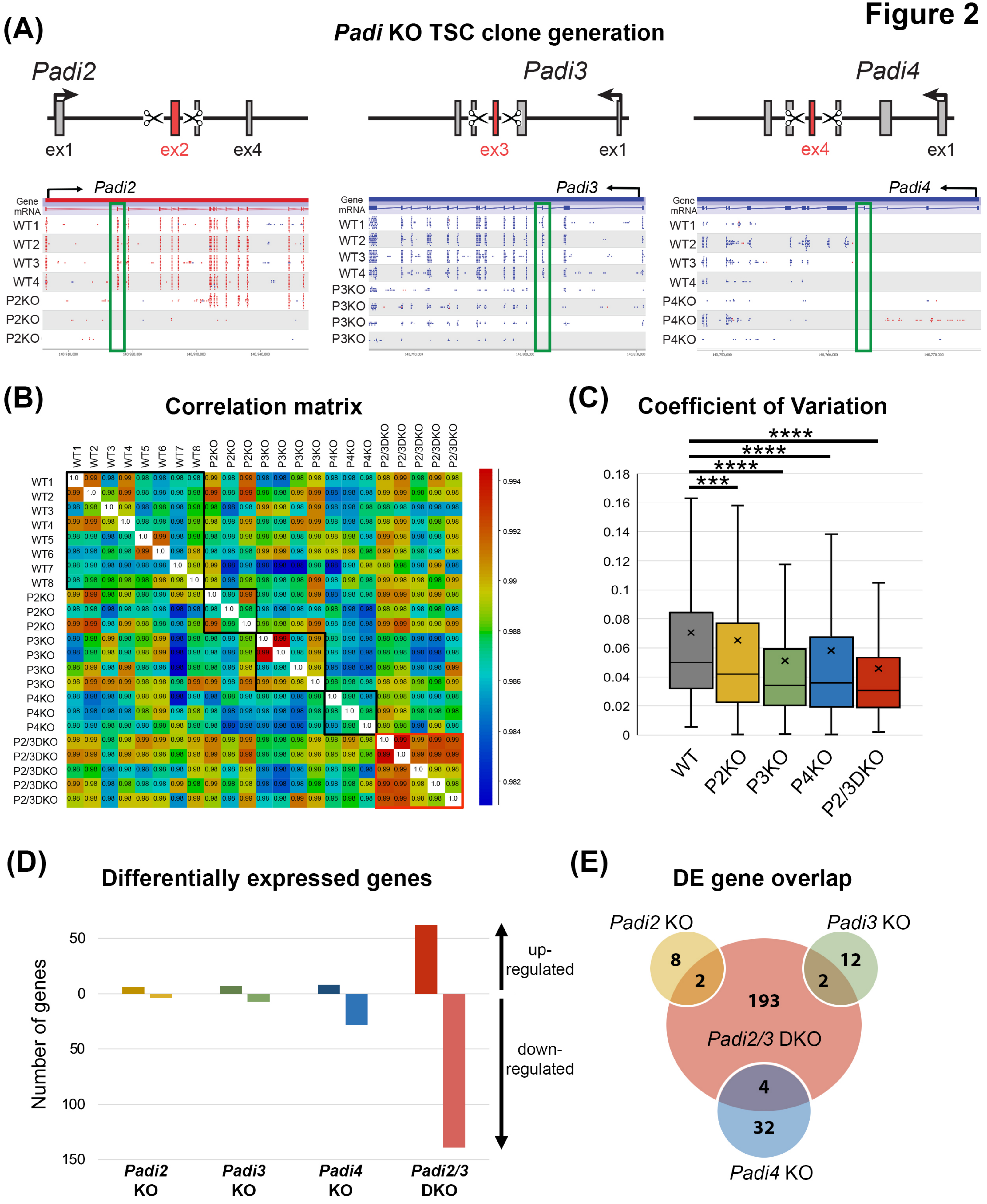
(A)
Padi KO TSC clone generation
Padi2
ex1
ex2
ex4
Padi3
ex3
ex1
Padi4
ex4
ex1
Gene
mRNA
WT1
WT2
WT3
WT4
P2KO
P3KO
P4KO
(B)
Correlation matrix
WT1 WT2 WT3 WT4 WT5 WT6 WT7 WT8
P2KO P2KO P2KO
P3KO P3KO P3KO P3KO
P4KO P4KO P4KO
P2/3DKO P2/3DKO P2/3DKO P2/3DKO P2/3DKO
0.994
0.992
0.99
0.988
0.986
0.984
0.982
(C)
Coefficient of Variation
****
****
****
***
0.18
0.16
0.14
0.12
0.1
0.08
0.06
0.04
0.02
0
WT
P2KO
P3KO
P4KO
P2/3DKO
(D)
Differentially expressed genes
Number of genes
50
0
50
100
150
up-regulated
down-regulated
Padi2 KO
Padi3 KO
Padi4 KO
Padi2/3 DKO
(E)
DE gene overlap
Padi2 KO
Padi3 KO
8
2
12
2
193
Padi2/3 DKO
4
32
Padi4 KO

Figure 3

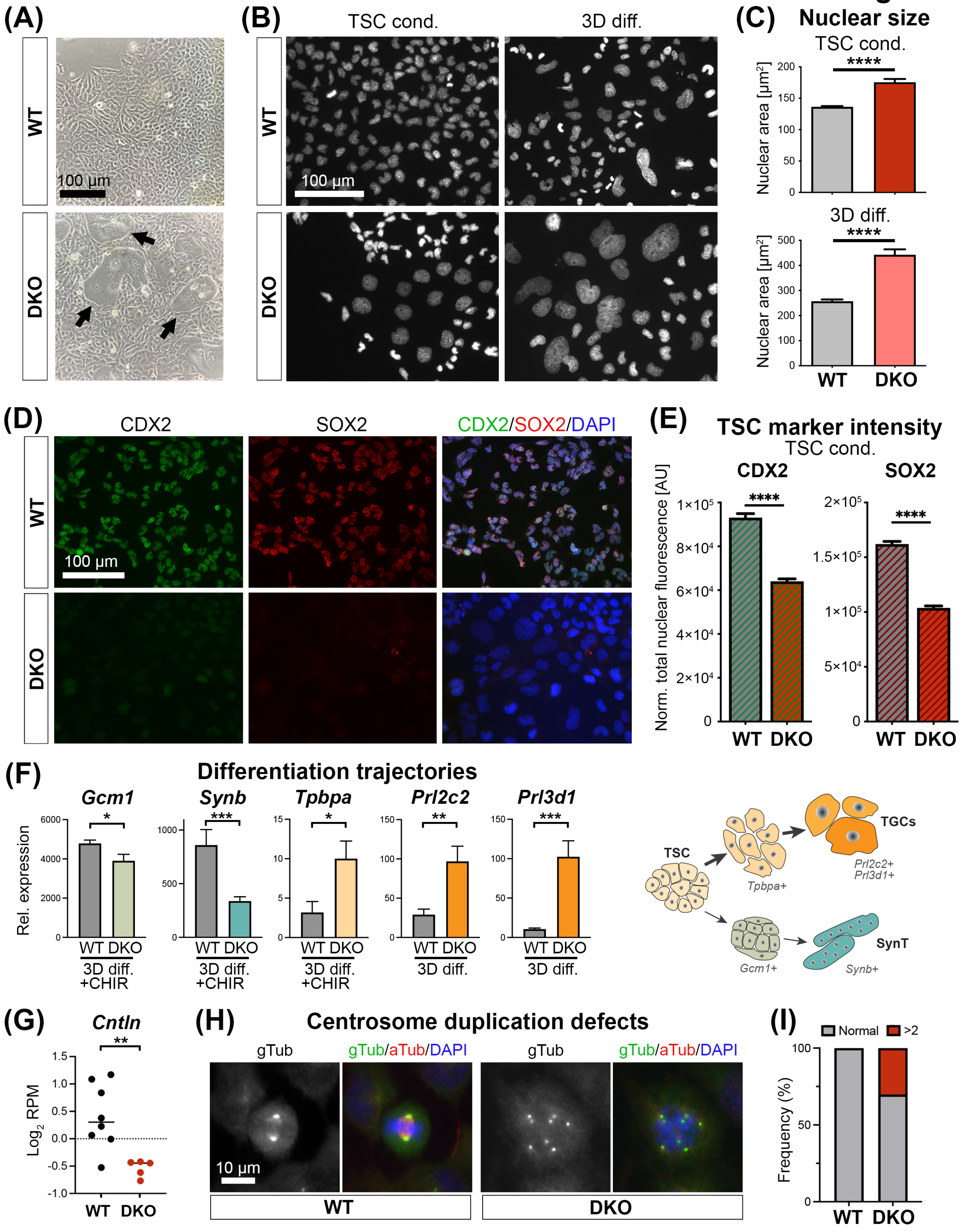

(A)
WT
DKO
100 µm
(B)
TSC cond.
3D diff.
WT
DKO
100 µm
(C)
Nuclear size
TSC cond.
Nuclear area [µm²]
****
3D diff.
****
WT
DKO
(D)
CDX2
SOX2
CDX2/SOX2/DAPI
WT
DKO
100 µm
(E)
TSC marker intensity
TSC cond.
CDX2
SOX2
Norm. total nuclear fluorescence [AU]
****
****
WT DKO
WT DKO
(F)
Differentiation trajectories
Gcm1
Synb
Tpbpa
Prl2c2
Prl3d1
Rel. expression
WT DKO
3D diff. +CHIR
3D diff.
TSC
Tpbpa+
TGCs
Prl2c2+
Prl3d1+
Gcm1+
SynT
Synb+
(G)
Cntln
Log₂ RPM
WT DKO
(H)
Centrosome duplication defects
gTub
gTub/aTub/DAPI
10 µm
WT
DKO
(I)
Normal
>2
Frequency (%)
WT DKO

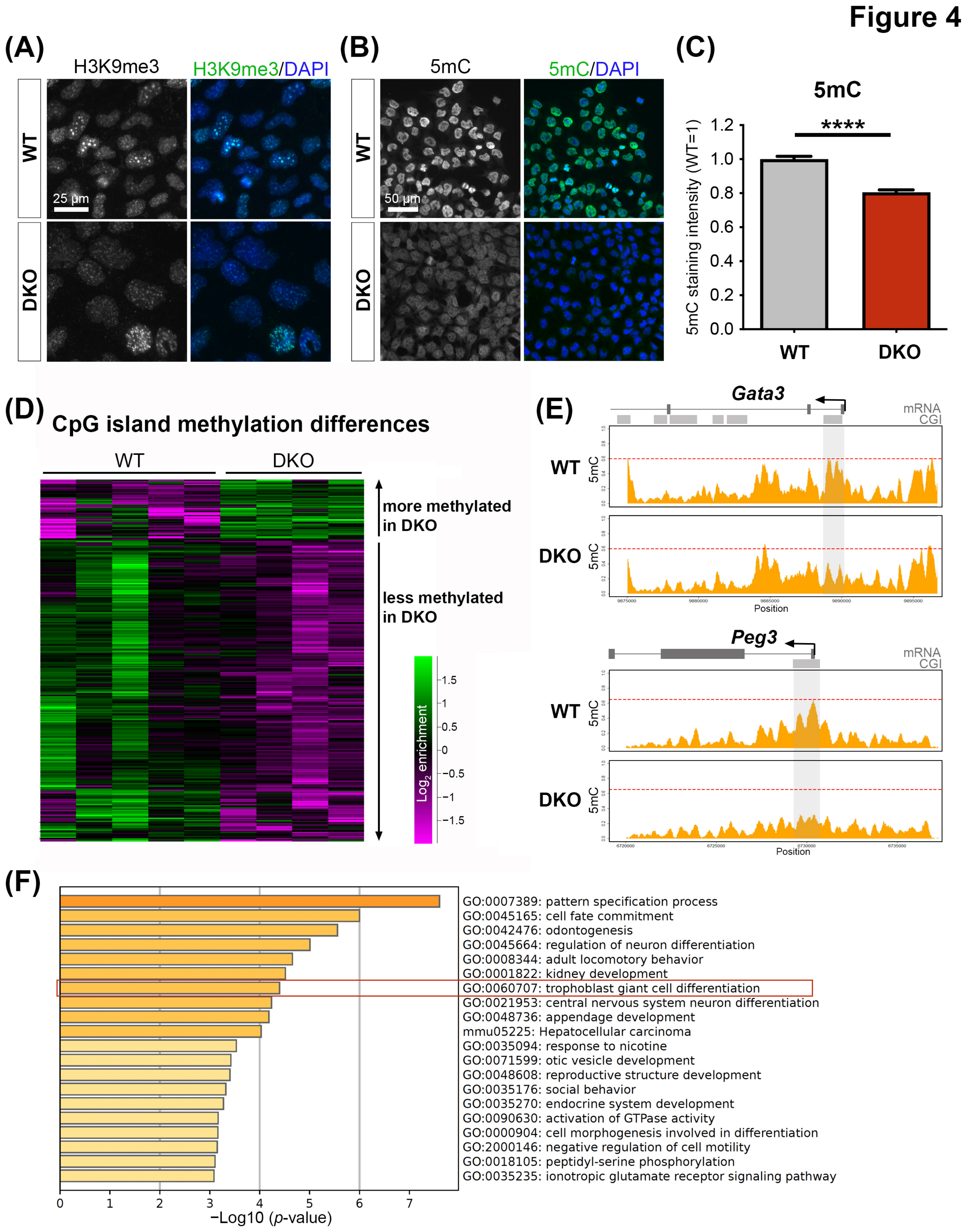
Figure 4
(A)
H3K9me3
H3K9me3/DAPI
WT
DKO
25 μm
(B)
5mC
5mC/DAPI
WT
DKO
50 μm
(C)
5mC
****
5mC staining intensity (WT=1)
1.2
1.0
0.8
0.6
0.4
0.2
0.0
WT
DKO
(D)
CpG island methylation differences
WT
DKO
more methylated in DKO
less methylated in DKO
Log2 enrichment
1.5
1
0.5
0
−0.5
−1
−1.5
(E)
Gata3
mRNA
CGI
WT
DKO
5mC
Position
Peg3
mRNA
CGI
WT
DKO
5mC
Position
(F)
GO:0007389: pattern specification process
GO:0045165: cell fate commitment
GO:0042476: odontogenesis
GO:0045664: regulation of neuron differentiation
GO:0008344: adult locomotory behavior
GO:0001822: kidney development
GO:0060707: trophoblast giant cell differentiation
GO:0021953: central nervous system neuron differentiation
GO:0048736: appendage development
mmu05225: Hepatocellular carcinoma
GO:0035094: response to nicotine
GO:0071599: otic vesicle development
GO:0048608: reproductive structure development
GO:0035176: social behavior
GO:0035270: endocrine system development
GO:0090630: activation of GTPase activity
GO:0000904: cell morphogenesis involved in differentiation
GO:2000146: negative regulation of cell motility
GO:0018105: peptidyl-serine phosphorylation
GO:0035235: ionotropic glutamate receptor signaling pathway
0
1
2
3
4
5
6
7
−Log10 (p-value)

Figure 5

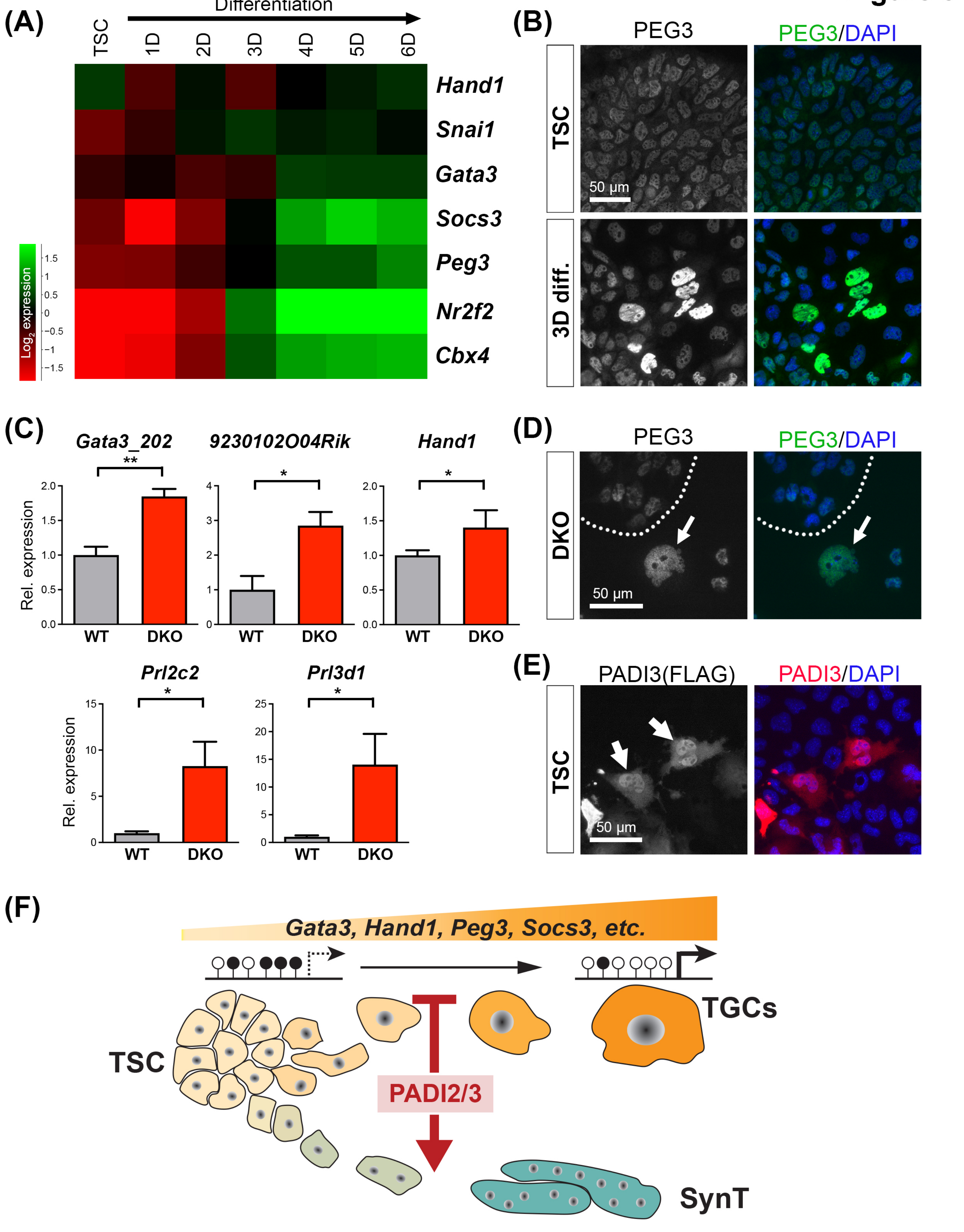

(A)
Differentiation
TSC
1D
2D
3D
4D
5D
6D
Hand1
Snai1
Gata3
Socs3
Peg3
Nr2f2
Cbx4
Log2 expression
1.5
1
0.5
0
−0.5
−1
−1.5
(B)
PEG3
PEG3/DAPI
TSC
3D diff.
50 μm
(C)
Gata3_202
9230102O04Rik
Hand1
Rel. expression
WT
DKO
Prl2c2
Prl3d1
(D)
PEG3
PEG3/DAPI
DKO
50 μm
(E)
PADI3(FLAG)
PADI3/DAPI
TSC
50 μm
(F)
Gata3, Hand1, Peg3, Socs3, etc.
TSC
TGCs
PADI2/3
SynT

Suppl. Figure S1

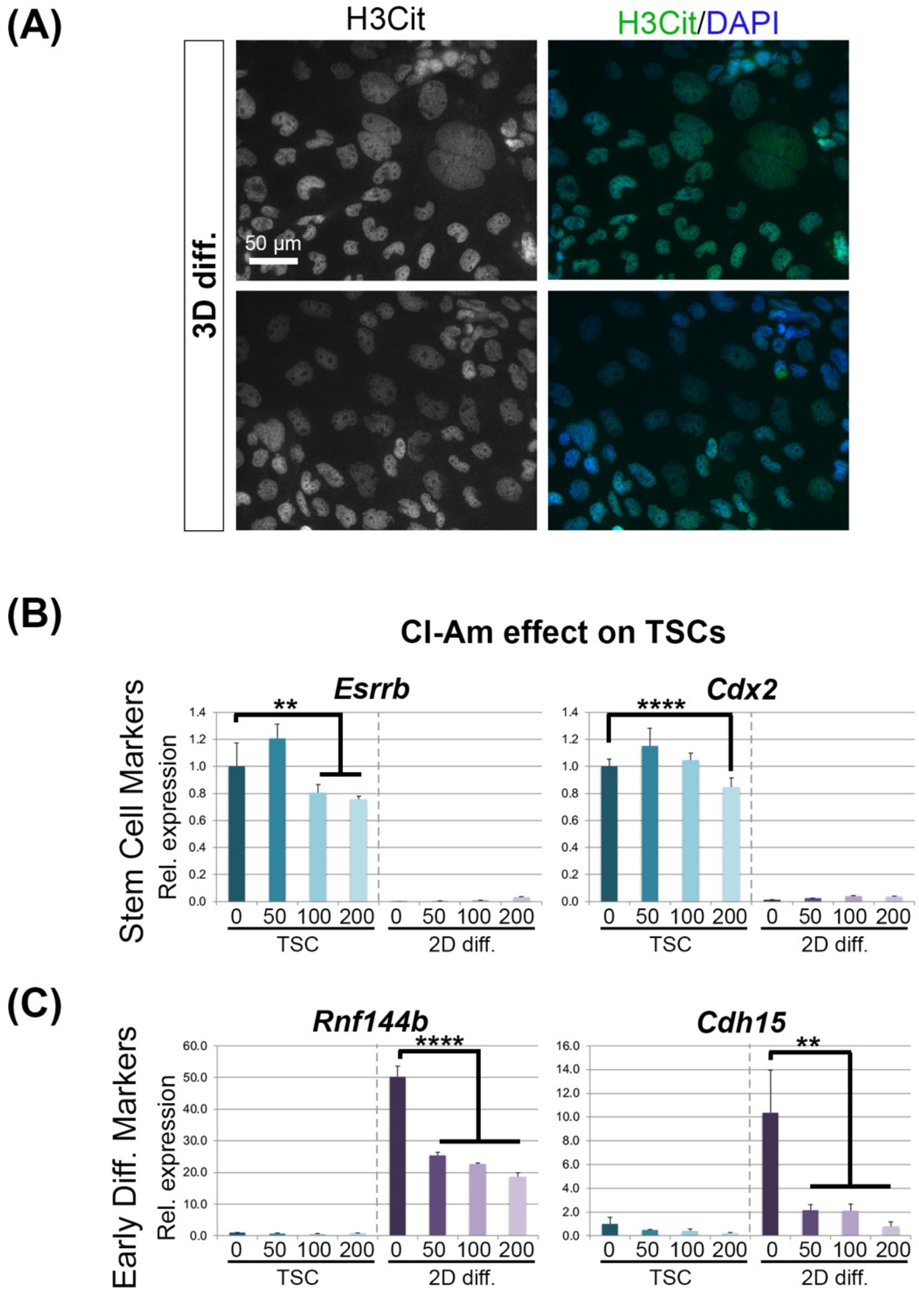

Supplementary Figure S1. Effect of PADI inhibition on TSC differentiation.

(**A**) Immunofluorescence staining against H3-R2/R8/R17 citrullination (H3Cit) in wild-type TSCs after 3 days (D) of differentiation. The representative images reveal that smaller, more stem-like cells exhibit higher levels of H3Cit staining than large, differentiated trophoblast giant cells. (**B, C**) RT-qPCR analysis of additional stem cell (in (B)) and early differentiation markers (in (C)) [42] in TSCs grown in stem cell conditions ("TSC") and after 2 days of differentiation ("2D diff.") in the presence of the indicated amounts of Chlor-Amidine. The down-regulation of early differentiation markers indicates that *Padi2/3* DKO TSCs cells have already progressed past this point of differentiation, as corroborated by the up-regulation of terminal differentiation marker *Prl2c2* (Figure 1F). Data are expressed relative to stem cell

control (0μM Cl-Am) and are displayed as mean +/− S.E.M. (n=3 independent replicates). Statistical analysis was conducted for the relevant combinations of markers and conditions (TSC conditions for stem cell markers, 2D diff. conditions for differentiation markers) by 2-way ANOVA with Dunnett's multiple comparisons test. ** $p<0.01$, **** $p<0.0001$.

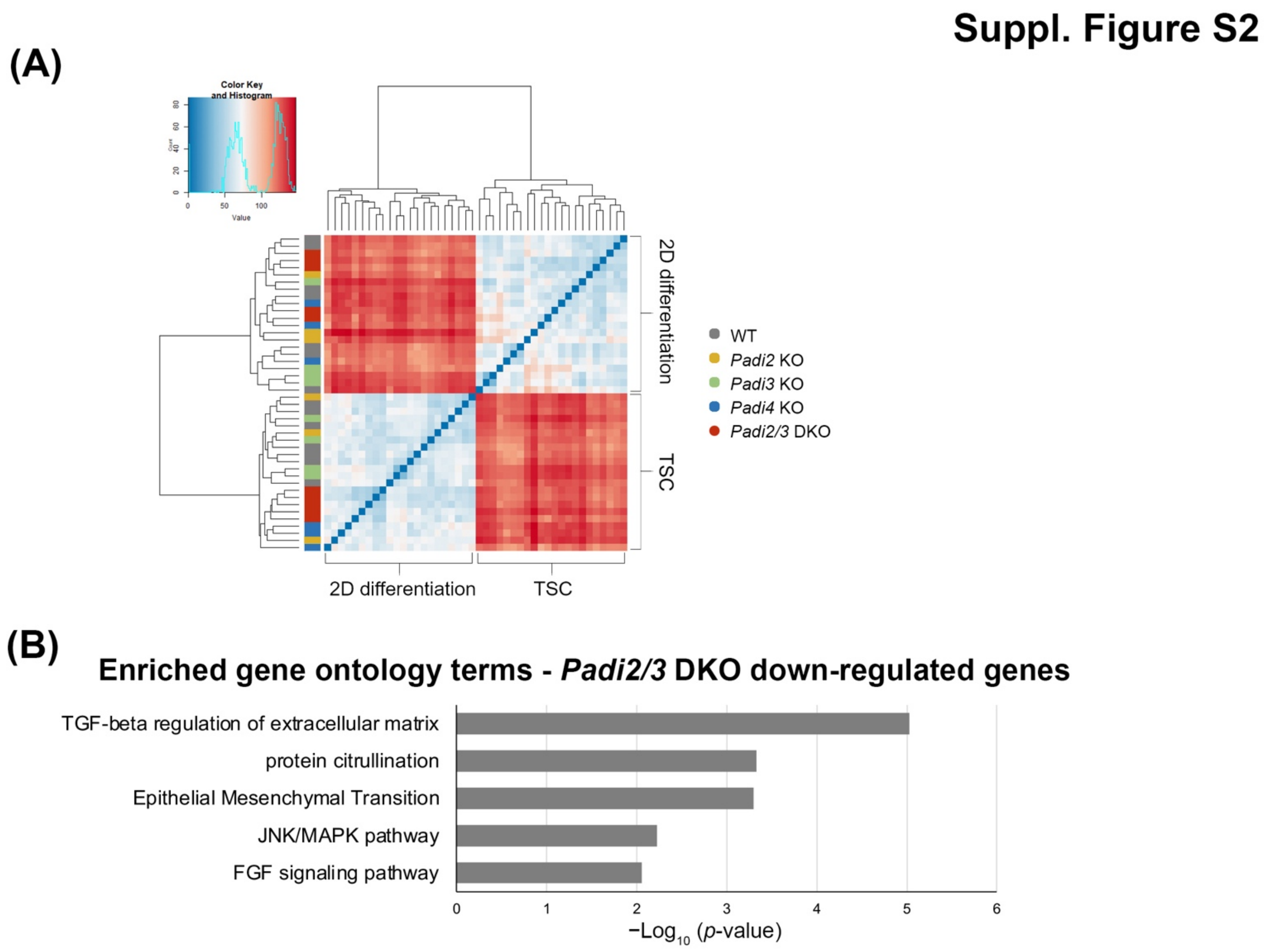

Supplementary Figure S2. Consequences of PADI deletion on TSC transcriptomes.

(**A**) Sample similarity heatmap of all single and double *Padi* KO clones in TSC conditions and after 2 days (D) of differentiation. The sample distribution shows that the primary segregation of samples is by stage of differentiation, unrelated to genotype. (**B**) EnrichR gene ontology analysis on genes down-regulated in *Padi2/3* double knockout (DKO) TSCs.

Suppl. Figure S3

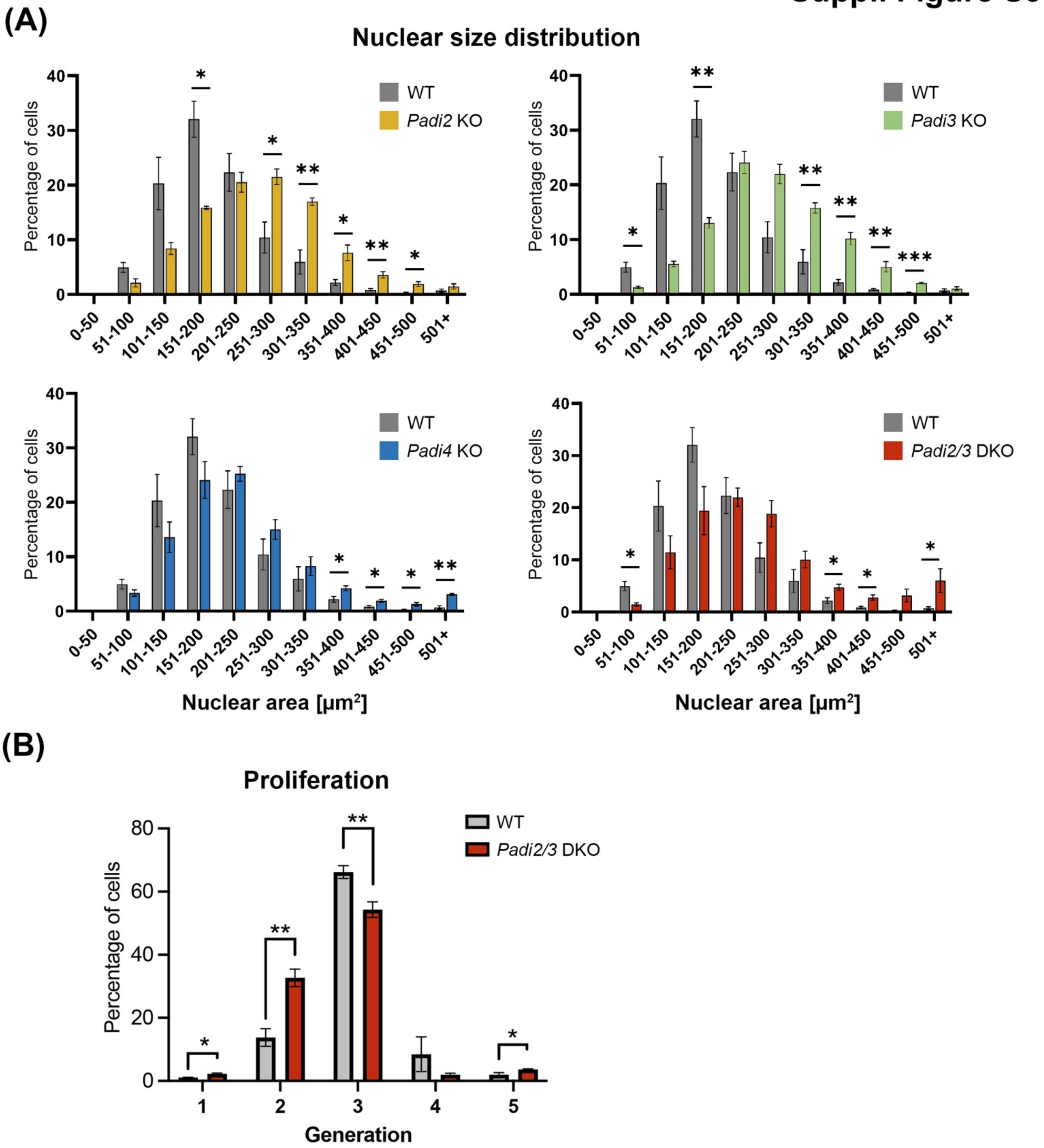

Supplementary Figure S3. ***Padi2/3* ablation causes TSCs to become larger and less proliferative.**
(**A**) Nuclear size distribution profiles of *Padi2* KO, *Padi3* KO, *Padi4* KO and *Padi2/3* DKO TSCs compared to wild-type (WT) control cells. The fraction of enlarged cells is evident in all single KOs but is most pronounced in the *Padi2/3* DKO. N≥3 independent clones per genotype. Statistical analysis by one-way ANOVA (* p<0.05, ** p<0.01). (**B**) Proliferation rate analysis of WT compared to *Padi2/3* DKO TSCs as determined upon CFSE incorporation. *Padi2/3* DKO TSCs contained a higher fraction of cells in

generations 1 and 2 whereas fewer had progressed to generation 3, indicative of an overall slower proliferation rate. Statistical analysis by Student's t-test (equal variances) or Welch's T-test (unequal variances). * $p<0.05$, ** $p<0.01$.

Suppl. Figure S4

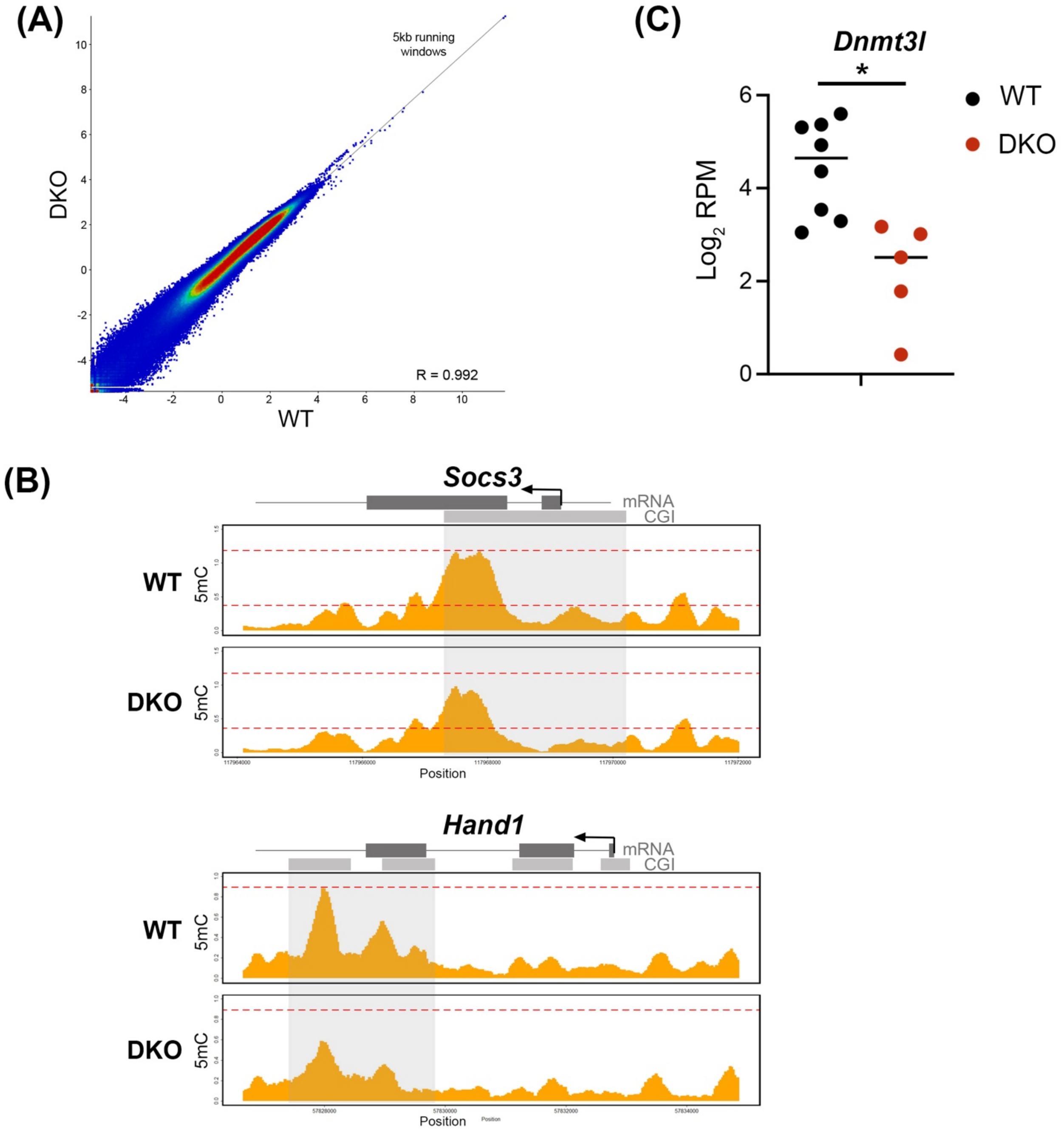

Supplementary Figure S4. ***Padi2/3*** **DKO TSCs exhibit reduced DNA methylation levels at key trophoblast gene loci.**

(**A**) Scatter plot of global DNA methylation profiles in WT compared to *Padi2/3* DKO TSCs as determined by read count enrichment across 5k running windows spaced 2.5kb apart. (**B**) Wiggle plots of 5mC enrichment across the *Socs3* and *Hand1* loci. Gene structure (exons) and CGIs are displayed on top of the graphs. The differentially methylated CGIs are shaded. The red dotted lines demarcate the level of maximal (*Hand1*) and maximal and minimal (*Socs3*) enrichment in WT cells, and are drawn at the corresponding levels in the DKO graph to help visualize the reduction in 5mC enrichment. (**C**)

Expression levels of *Dnmt3l* in WT and *Padi2/3* DKO TSCs as determined by RNA-seq. Statistical analysis by unpaired Student's t-test (* $p<0.05$).

Suppl. Figure S5

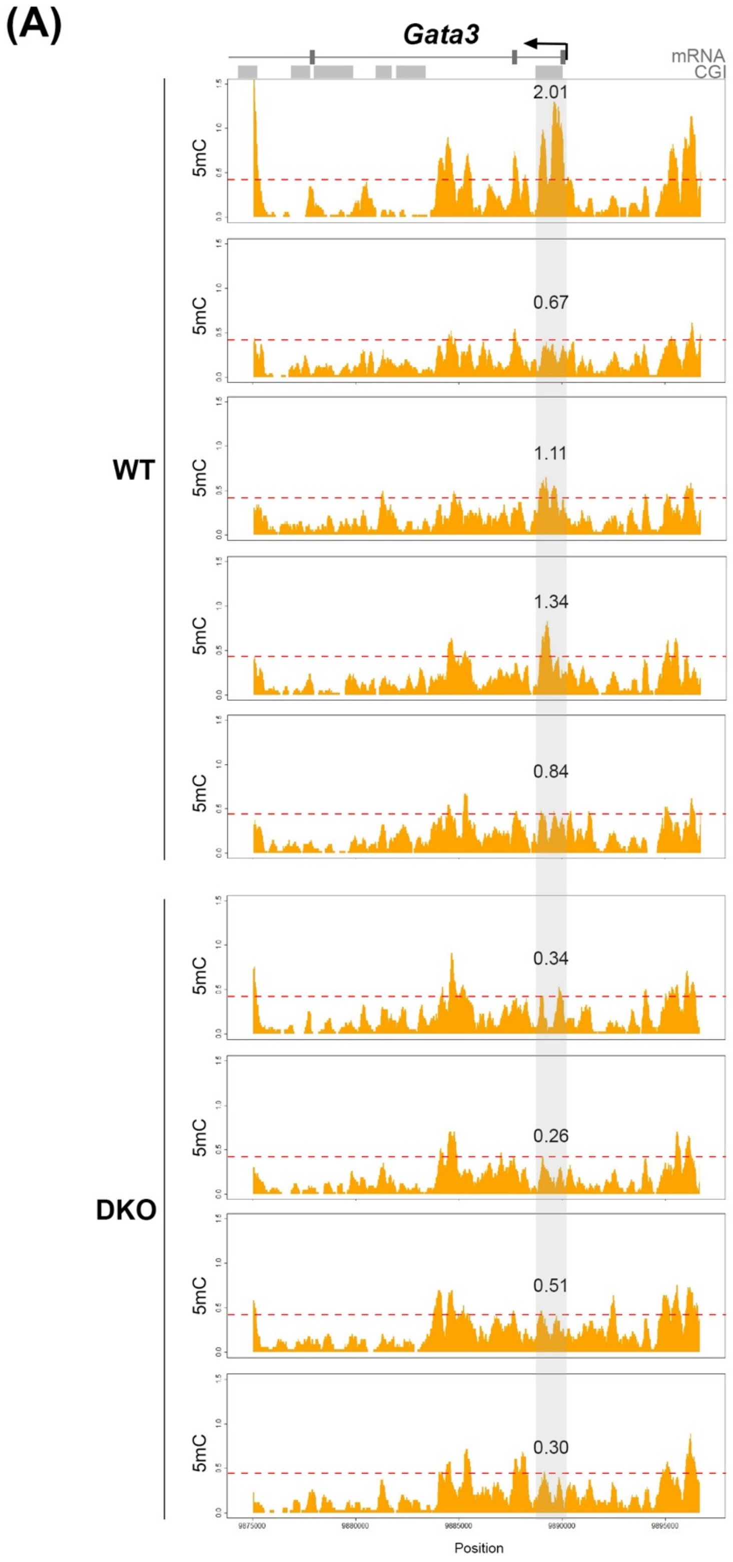

Supplementary Figure S5. DNA methylation differences are comparable between independently derived cell clones.

(**A**) Wiggle plots of 5mC enrichment across the *Gata3* locus as shown in Figure 4E, displayed for each individual single cell-derived WT and *Padi2/3* DKO TSC clone. Gene structure (exons) and CGIs are

displayed on top of the graphs. The differentially methylated CGI is shaded. The red dotted line is drawn at equivalent enrichment values to help visualize the reduction in 5mC enrichment in *Padi2/3* DKO cells. Log_2-fold enrichment values normalized to library size across the differentially methylated CGI are provided.

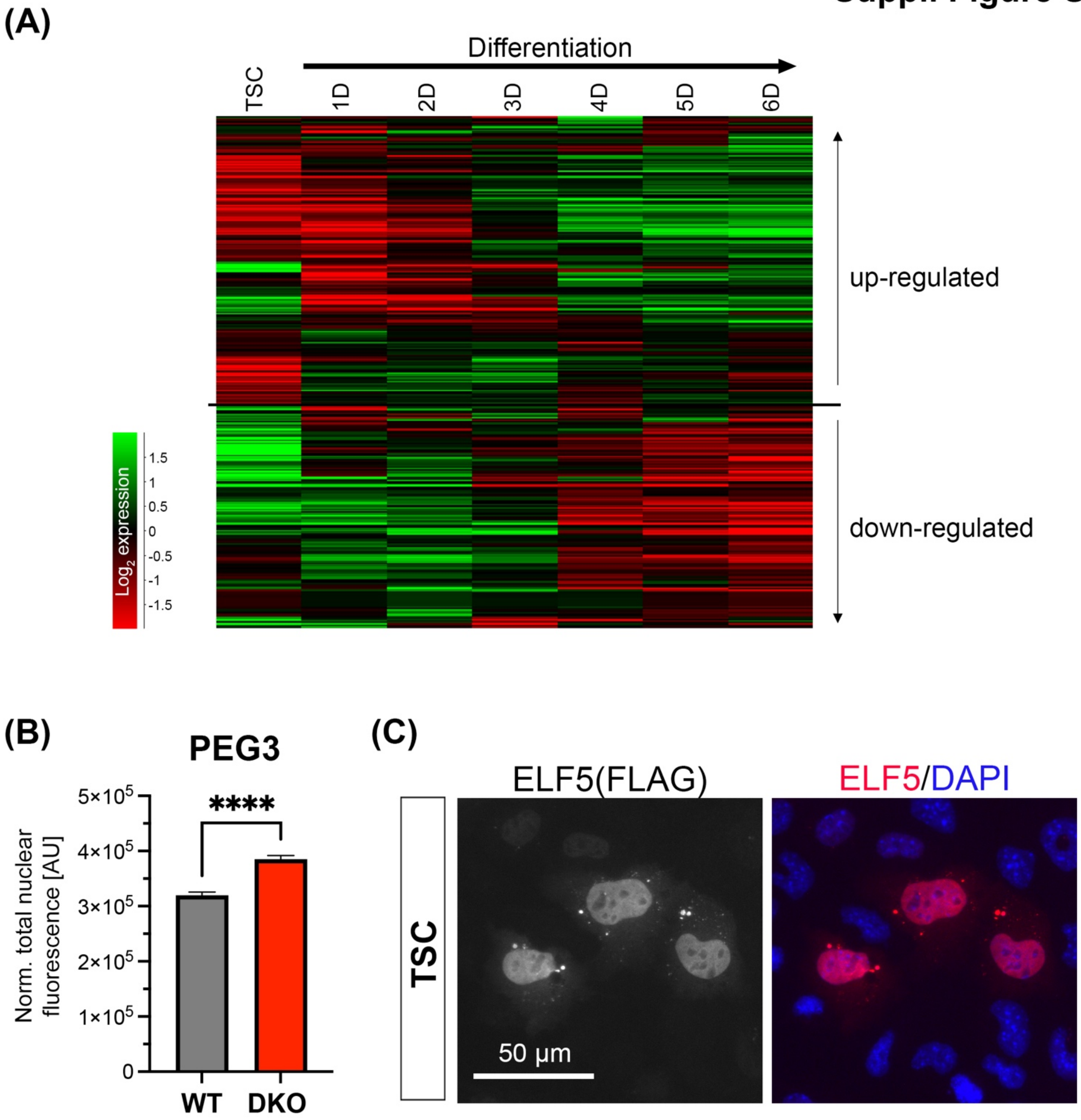

Supplementary Figure S6. Regulation of genes associated with hypomethylated CGIs.

(**A**) Expression dynamics of genes that are close to (within 2kb) hypomethylated CGIs in *Padi2/3* DKO cells across a TSC differentiation time course. (**B**) Quantification of PEG3 immunofluorescence staining intensities as shown in Figure 5D across the cell populations of WT and *Padi2/3* DKO TSCs grown in stem cell conditions. AU = arbitrary units. Statistical analysis by unpaired Student's t-test (**** p<0.0001; n=4 independent clones per genotype with >150 cells assessed each). (**C**) Immunostaining for FLAG-tagged ELF5 in TSCs as transfection and staining control of the *Padi3*-FLAG expression construct used in Figure 5E. ELF5 is a transcription factor with predominantly nuclear localization.
